# Same geometry, different dynamics: what uncontrolled manifold (UCM) analysis misses about motor control

**DOI:** 10.1007/s00221-026-07404-9

**Published:** 2026-09-30

**Authors:** Madhur Mangalam, Nick Stergiou

**Affiliations:** 1 Department of Biomechanics, University of Nebraska at Omaha, Omaha, 68182 USA; 2 Department of Physical Education & Sport Science, Aristotle University, Thessaloniki, 57001 Greece

**Keywords:** Biomechanics, Motor control, Movement variability, Motor learning, Temporal structure, Uncontrolled manifold

## Abstract

Uncontrolled Manifold (UCM) analysis separates the variability of multi-joint motion into a part that affects the task goal (task-relevant) and a part that does not (task-irrelevant), using the geometric relationship—the task Jacobian—between joint motion and task outcome. The resulting partition has been interpreted as evidence of neural control strategies that stabilize task goals while permitting flexibility in execution. UCM is conventionally reported as time-averaged variance indices, and this time-averaging step discards the temporal structure of movement variability. Using a three-joint planar reaching model, we generate variability with three temporal structures (periodic oscillation, white noise, and pink noise) matched in spatial covariance, and find that the time-averaged geometric indices—the ratio $V_{\text {UCM}}/V_{\text {ORT}}$ and the *Z*-transformed synergy index $\Delta V_z$—remain statistically indistinguishable for white and pink noise, whereas temporal indices computed on the same trials (lag-1 autocorrelation, detrended-fluctuation scaling, spectral slope) show large effect sizes. This double dissociation confirms that time-averaged summaries remain blind to temporal correlations. The projection operation retains temporal information in the single-trial and time-resolved components, yet the meaning of that information for motor control remains open: the mapping from a temporal property of a projected series to a specific control process is itself unestablished, so applying time-series methods to the projections opens a fresh interpretive problem. UCM therefore identifies whether coordination is structured by task constraints while leaving the generating processes undetermined. Establishing this boundary is the contribution of the present work: geometric partitioning yields a spatial account of coordination whose temporal origins lie beyond the method and call for a dedicated dynamical theory.

## Introduction

Motor variability was historically considered “noise” that needed to be minimized or averaged away (Fitts 1954; Schmidt 1975; Slifkin et al. 2000; Woodworth 1899). Analyzing motor variability as a signal—structured patterns that reveal control principles—has fundamentally changed how we study motor control (Bernstein 1967; Latash 2012, 2021; Profeta and Turvey 2018; Turvey 1990, 2007; Turvey and Fonseca 2014; van Beers 2009). Within the family of variance decomposition methods, Uncontrolled Manifold (UCM) analysis has become the most dominant framework for inferring coordination strategies from motor variability data (Latash et al. 2007; Scholz and Schöner 1999). UCM divides the variation in basic variables such as joint angles, muscle activations, and forces into components that affect task-level performance variables and those that do not. The UCM method proceeds by examining the task Jacobian, which defines the geometric relationship between these variables. The fundamental premise is that higher variance within the UCM—the set of joint configurations that leave the task outcome unchanged—compared to variance orthogonal to it (the directions that do change it; $V_{\text {UCM}}\!>\!V_{\text {ORT}}$) reflects control organization that stabilizes task outcomes while permitting flexibility in execution (Latash et al. 2002; Scholz and Schöner 1999). This interpretation has driven extensive research characterizing principles of control organization: task-level stabilization, exploitation of redundancy, and synergy formation.

UCM has been applied across motor domains—multi-finger force production (Latash et al. 2001; Scholz et al. 2003; Tillman and Ambike 2018), postural control (Reimann and Schöner 2017; Scholz and Schöner 1999), walking and running (Black et al. 2007; Decker et al. 2012; Papi et al. 2015), pointing (Domkin et al. 2005; Scholz et al. 2000), reaching (Tseng et al. 2002), and grasping (Shim et al. 2004)—in each case, $V_{\text {UCM}}/V_{\text {ORT}}$ ratios interpreted as evidence for or against flexible neural control, with values above unity taken as goal-directed stabilization and values near unity as its absence (Latash et al. 2002; Scholz and Schöner 1999). The framework has been extended to test predictions about performance-stabilizing synergies (Latash et al. 2007; Latash 2021, 2024a, b), redundancy resolution (Berret et al. 2011; Campolo et al. 2013; Latash et al. 2003), and task-specific flexibility (Freitas and Scholz 2009; Hsu and Scholz 2012; Kang et al. 2004; Scholz and Schöner 2014), across diverse tasks, establishing it as a standard method for inferring control strategies from movement variability.

What this standard UCM analysis captures and what it sets aside can be stated plainly. The method describes the *shape* of movement variability—which combinations of joint angles a performer lets vary and which they hold fixed—but not the *order* in which those variations unfold over time. Two performers who explore the same set of postures share an identical partition even if one drifts slowly and predictably while the other jitters at random, because that partition is reported as a single time-averaged number. The value it assigns them is the same, yet the control processes it implies are not. We aim to measure the size of this gap and to mark where a temporal account must take over.

**Fig. 1:**
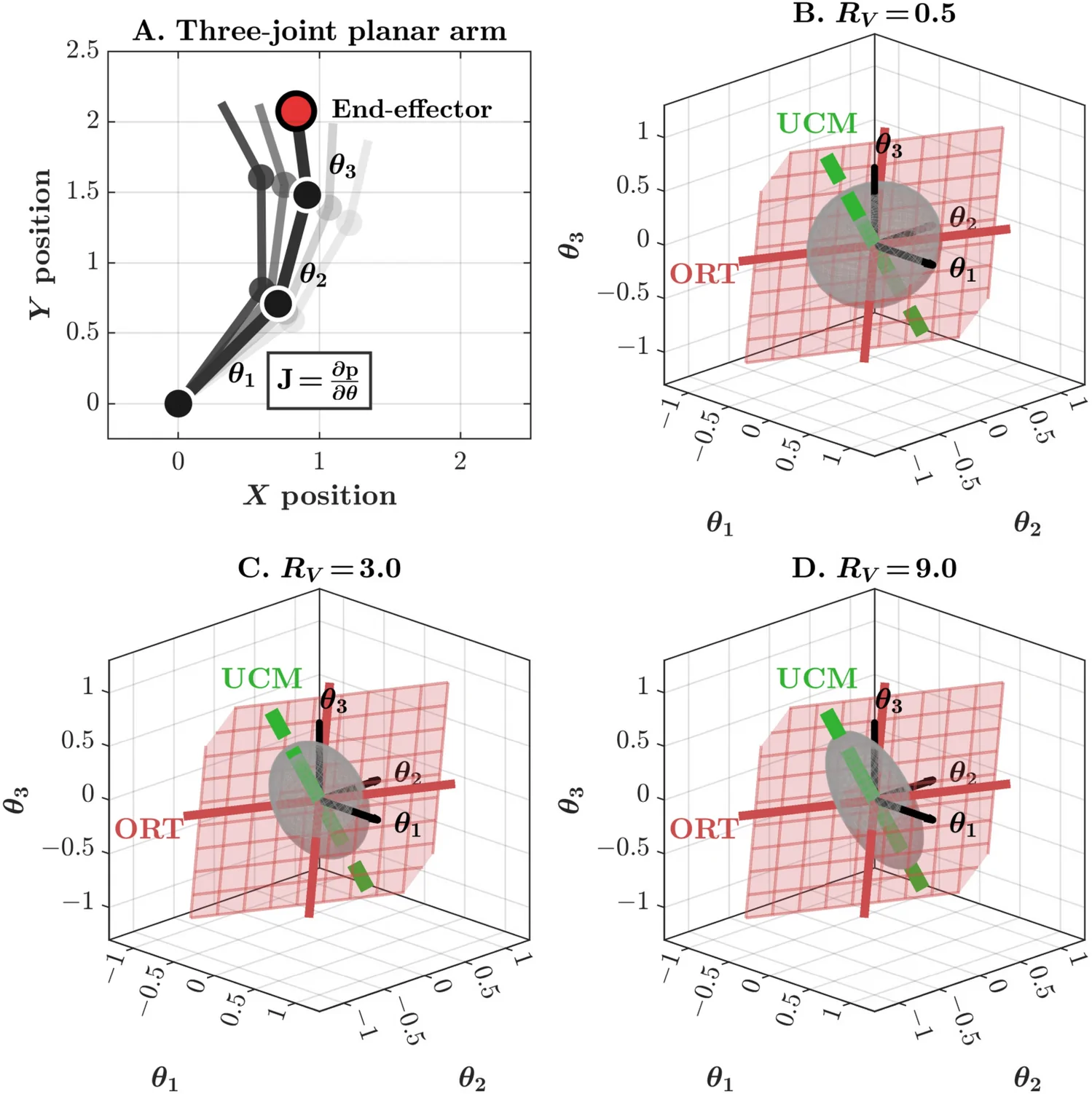
Uncontrolled Manifold analysis partitions joint-space variability based on geometric projection. (**A**) Three-joint planar arm performing a reaching task, wherein joint angles $\theta _1$, $\theta _2$, $\theta _3$ (elemental variables) determine end-effector position (performance variable). The task Jacobian $\textbf{J}\!=\!\partial \textbf{p} / \partial \boldsymbol{\theta }$ defines the uncontrolled manifold (UCM, green dashed line)—the one-dimensional null space—and orthogonal subspace (ORT, red solid lines and plane)—the two-dimensional range space. Ghost trajectories illustrate movement variability. (**B–D**) UCM variance decomposition in three-dimensional joint angle space ($\theta _1$, $\theta _2$, $\theta _3$). Gray ellipsoids represent variance distribution; green dashed line shows the UCM direction (1D null space); red solid lines show the two ORT basis directions spanning the 2D range space; red semi-transparent plane visualizes the ORT subspace. Black arrows indicate joint angle coordinate axes. (**B**) When $R_V\!=\!V_{\text {UCM}}/V_{\text {ORT}}\!<\!1$, the variance that affects the task performance (ORT plane) exceeds the variance that does not affect the task performance (UCM line), indicating poor coordination. The ellipsoid is flattened along the UCM direction (pancake-shaped). (**C**) When $R_V\!>\!1$, variance is preferentially organized along the UCM direction, indicating selective stabilization of task-relevant dimensions. The ellipsoid elongates along the UCM line (cigar-shaped). (**D**) Very high ratios ($R_V\!\gg \!1$) indicate strong synergistic control with task-irrelevant variability substantially exceeding task-relevant variability. The ellipsoid is highly elongated along the UCM direction.

Notably, UCM analysis is fundamentally a geometric decomposition. At each time point, variance is projected onto subspaces defined by the Jacobian matrix $\textbf{J}\!=\!\partial g / \partial \boldsymbol{\theta }$, where $g(\boldsymbol{\theta })$ maps elemental variables $\boldsymbol{\theta }$—measurements of joint angles, muscle activations, or forces, depending on the task—to performance outcomes, so that $\textbf{J}$ relates small changes in $\boldsymbol{\theta }$ to changes in performance. The null space $\mathcal {N}(\textbf{J})$ comprises configurations that do not alter task performance to first order, while the range space $\mathcal {R}(\textbf{J})$ contains directions that do. Variance components are computed as:

1 $$\begin{aligned} V_{\text {UCM}}(t)&= \frac{1}{n_{\text {trials}} \cdot \dim (\mathcal {N})} \sum _{i=1}^{n_{\text {trials}}} \Vert \textbf{P}_{\mathcal {N}} \Delta \boldsymbol{\theta }_i(t) \Vert ^2, \end{aligned}$$

2 $$\begin{aligned} V_{\text {ORT}}(t)&= \frac{1}{n_{\text {trials}} \cdot \dim (\mathcal {R})} \sum _{i=1}^{n_{\text {trials}}} \Vert \textbf{P}_{\mathcal {R}} \Delta \boldsymbol{\theta }_i(t) \Vert ^2, \end{aligned}$$

where $\textbf{P}_{\mathcal {N}}$ and $\textbf{P}_{\mathcal {R}}$ are projection operators, and $\Delta \boldsymbol{\theta }_i(t)$ is the deviation of trial *i* from the mean trajectory at time *t*. Fig. 1 makes this concrete for a three-joint planar arm reaching to a target: the same trial-to-trial wobble in the joints is sorted into the part that moves the hand off target and the part that shuffles the joints without moving the hand. This formulation uses the Jacobian linearization, which is standard in classical UCM analysis and valid for small deviations from the nominal configuration. The geometric interpretation is straightforward: higher variance within the UCM relative to the orthogonal subspace indicates that variability is preferentially organized in directions that preserve task performance. However, the variance indices conventionally derived from this decomposition—once averaged over time—take the same value whether such variance patterns arise from temporally structured control processes (e.g., periodic oscillations, long-range correlations) or temporally unstructured noise, as we demonstrate here. The projection itself transmits temporal structure to the time-resolved components $V_{\text {UCM}}(t)$ and $V_{\text {ORT}}(t)$ and to the per-trial projections; the time-averaging step collapses it. What the temporal behavior of those projected components reveals about the control process is a further question the present work raises and leaves for dedicated theoretical treatment.

**Fig. 2:**
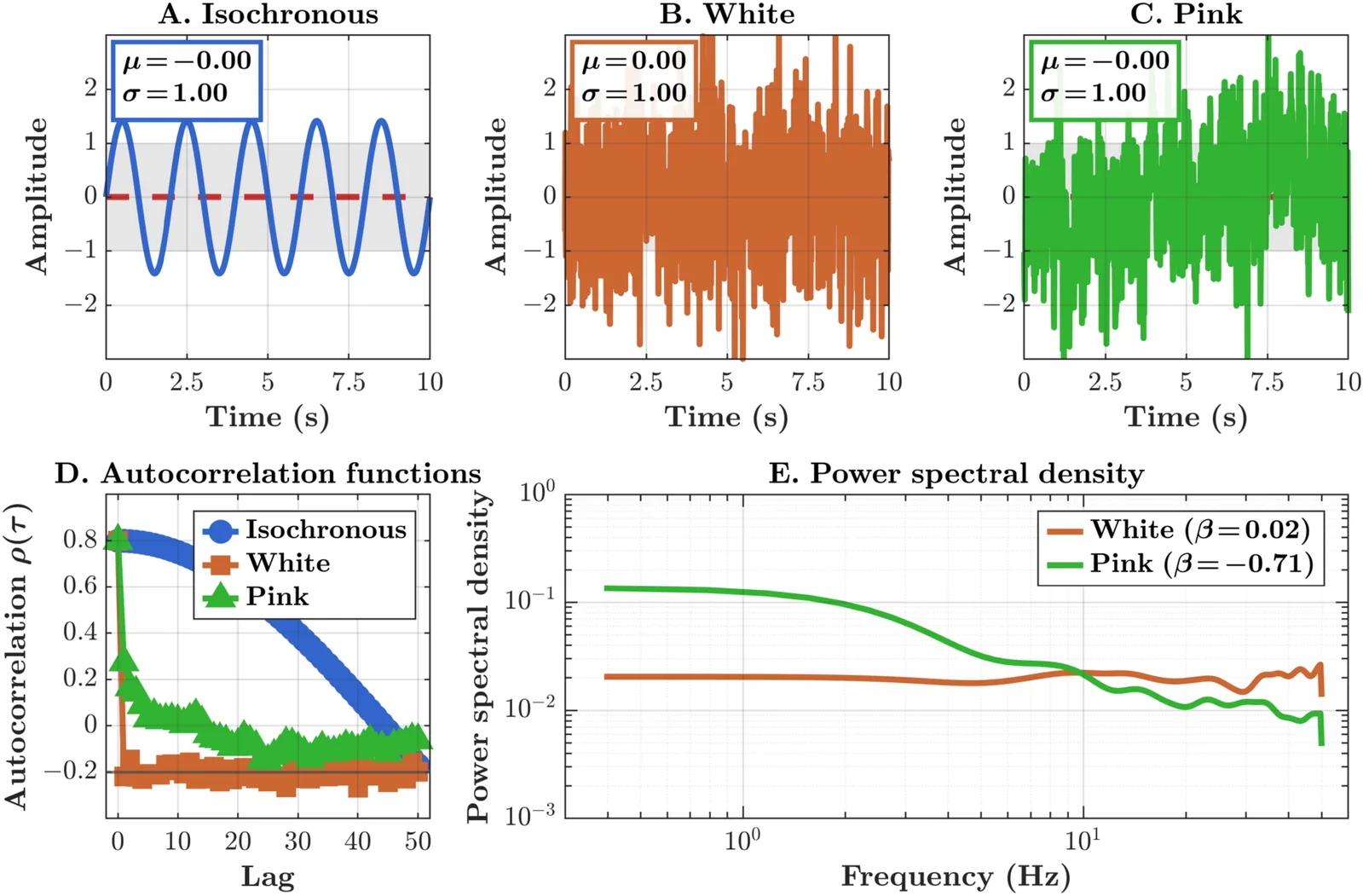
Signals with identical mean and standard deviation can exhibit fundamentally different temporal structures that time-averaged variance summaries cannot distinguish. (**A–C**) Time series with identical statistical moments across three conditions: (**A**) Isochronous exhibits deterministic periodic oscillations at *0.5* Hz. (**B**) White noise exhibits temporally uncorrelated Gaussian fluctuations. (**C**) Pink noise exhibits *1/f* power spectral density with long-range temporal correlations. All signals normalized to $\mu \!=\!0$ and $\sigma \!=\!1$ (shown in annotation boxes). Red dashed line indicates mean; gray shaded region indicates *± 1* standard deviation. (**D**) Autocorrelation functions reveal distinct temporal dependencies: isochronous shows strong periodic structure with $\rho (1)\!=\!0.85$, white noise shows zero correlation with $\rho (1)\!\approx \!0$, and pink noise shows slowly decaying positive correlation with $\rho (1)\!=\!0.23$. (**E**) Power spectral density in log–log coordinates. The isochronous signal is characterized by a dominant oscillatory frequency (not shown). White noise (orange) exhibits an approximately flat broadband spectrum ($\beta \!=\!0.02$), whereas pink noise (green) exhibits a negatively sloped spectrum ($\beta \!=\!-0.71$) consistent with long-range temporal correlations.

The formulation above operates independently at each time point. Time-varying UCM analyses exist; they track how the *spatial* organization of variance changes across movement phases—how ratios evolve from initiation to completion (Martin et al. 2019), how the UCM subspace reorients during practice (Domkin et al. 2005; Yang et al. 2007), or how it shifts during anticipation of perturbations (Goodman and Latash 2006; Olafsdottir et al. 2005; Shim et al. 2005). They leave aside the *temporal dependencies within* the variance, that is, how variance at one moment relates to variance at later moments. Two methods are informative for the short series typical of motor data: autocorrelation, which quantifies persistence of deviations (Box et al. 2015; Chatfield and Xing 2019), and detrended fluctuation analysis (DFA), which captures long-range dependence robustly under nonstationarity (Peng et al. 1994; Kantelhardt et al. 2002). Power spectral density provides a frequency-domain view (Percival and Walden 1993); its slope is unreliable in short series, and we treat it as corroborative evidence. Put simply, these methods ask not how large the fluctuations are but how each moment relates to the next. Fig. 2 makes the core point concretely: three signals—isochronous, white, and pink—normalized to identical mean and variance nonetheless differ sharply in autocorrelation and spectral slope. Any summary statistic that depends on the first two moments alone assigns them the same value. When such structures are embedded in motor variability, the time-averaged UCM indices also assign the same value, because collapsing the partition over time discards temporal ordering.

These temporal structures correspond to mechanistically distinct control architectures. White noise—uncorrelated fluctuations with a flat spectrum—may reflect independent perturbations such as sensory noise (Faisal et al. 2008; Harris and Wolpert 1998), motor-unit recruitment variability (Jones et al. 2002), or measurement error (Winter 2009). Pink noise ($S(f)\!\propto \!1/f$) is a signature of scale-free dynamics coordinated across timescales (Chen et al. 2001; Diniz et al. 2011; Goldberger et al. 2002; Stergiou 2018) and appears in postural sway (Amoud et al. 2007; Błaszczyk and Klonowski 2001; Collins and De Luca 1995), finger force fluctuations (Deutsch and Newell 2003; Sosnoff et al. 2009; Vaillancourt and Newell 2003), and inter-stride intervals (Hausdorff 2007; Jordan et al. 2006; Terrier and Dériaz 2011). Deterministic periodic dynamics reflect rhythmic oscillators or entrainment (Haken et al. 1985; Hancock et al. 2025; Kelso 1995; Schöner 1990); exponential autocorrelation decay reveals feedback-mediated error correction with sensorimotor-loop delays (Diedrichsen et al. 2010; Shadmehr et al. 2010; Scott 2004; Todorov and Jordan 2002; Todorov 2004); and long-range correlations ($\alpha \!>\!0.5$) indicate persistent memory across timescales (Kello et al. 2010; Mangalam et al. 2023; Stephen and Dixon 2011; Van Orden et al. 2003). These features are orthogonal to the geometric partition: two systems with identical $V_{\text {UCM}}/V_{\text {ORT}}$ ratios may arise from entirely different processes—one actively correcting errors via feedback, the other passively accumulating biomechanically filtered noise.

Despite three decades of application, no study has systematically examined whether the conventional time-averaged indices can distinguish temporal structures corresponding to mechanistically distinct control processes. The gap matters because mechanistic claims throughout the literature depend on it. Theoretical tests require confirming that variance patterns exhibit predicted temporal dynamics—e.g., exponential relaxation with $\tau \!=\!B/K$—yet this is rarely reported alongside standard UCM summaries (Scholz et al. 2000; Tseng et al. 2002; Yang et al. 2007). The same holds for separating active neural coordination from passive mechanical compensation: multi-finger force studies inferred active stabilization from high ratios (Latash et al. 2001; Scholz et al. 2003; Shim et al. 2004) without testing for feedback-mediated autocorrelation (time constants of 100–200 ms; Scott, 2004; Todorov and Jordan, 2002; Todorov, 2004) versus passive biomechanical filtering (Prochazka et al. 1997; Rack and Westbury 1984); postural studies inferred active center-of-mass stabilization (Reimann and Schöner 2017; Scholz and Schöner 1999) without examining the multifractal signatures of adaptive control (Ihlen and Vereijken 2010; Kelty-Stephen et al. 2021); and clinical comparisons reported ratio differences (Black et al. 2007; Papi et al. 2015; Park et al. 2014; Ricotta and Latash 2021) without determining whether they reflect lost neural control or compensatory passive mechanics. In each case, the mechanistic interpretation rests on geometric evidence alone and remains untested at the level of the underlying generating process.

The present study addresses this gap through simulation, which is necessary because demonstrating the time-averaged indices’ insensitivity requires parametric control over temporal dynamics while holding the spatial variance structure constant— a condition infeasible in biological systems, where the two are inextricably coupled. We simulate trial-to-trial variability in a static multi-joint posture with three temporal structures (isochronous, white, and pink) while matching task- and joint-space variability, then apply UCM together with temporal characterization (autocorrelation, PSD, DFA) to quantify how far the time-averaged indices stay invariant to temporal structure and to examine what the projected components carry and what interpreting them would require.

Our central hypothesis is that the conventional time-averaged UCM indices will remain substantially insensitive to the temporal structure of motor variability, producing similar time-averaged $V_{\text {UCM}}/V_{\text {ORT}}$ values across conditions that differ fundamentally in autocorrelation, spectral, and long-range correlation properties. If confirmed, this establishes that the standard scalar UCM summaries characterize geometric coordination patterns while leaving the temporal dynamics of the underlying control processes undetermined. The projection retains temporal information in the single-trial and time-resolved components, yet the interpretive value of that information stays open, because the mapping from a temporal property of a projected series to a specific control process awaits its own justification. The implications extend to theoretical frameworks that rely on geometric interpretations of motor coordination, whose temporal predictions lie beyond the reach of the time-averaged indices.

## Methods

### Kinematic model

We simulated trial-to-trial variability in a planar three-joint arm configuration consisting of three rigid segments with lengths $L_1\!=\!1.0$, $L_2\!=\!0.8$, and $L_3\!=\!0.6$ (arbitrary units). The joint angles $\boldsymbol{\theta }\!=\![\theta _1, \theta _2, \theta _3]^T$ were defined relative to a fixed horizontal reference frame, where $\theta _1$ represents shoulder angle, $\theta _2$ elbow angle relative to $\theta _1$, and $\theta _3$ wrist angle relative to $\theta _1\!+\!\theta _2$. The forward kinematics mapping joint configuration to end-effector position $\textbf{p}\!=\![p_x, p_y]^T$ is:

3 $$\begin{aligned} p_x(\boldsymbol{\theta })&= L_1 \cos (\theta _1) + L_2 \cos (\theta _1 + \theta _2) + L_3 \cos (\theta _1 + \theta _2 + \theta _3), \end{aligned}$$

4 $$\begin{aligned} p_y(\boldsymbol{\theta })&= L_1 \sin (\theta _1) + L_2 \sin (\theta _1 + \theta _2) + L_3 \sin (\theta _1 + \theta _2 + \theta _3). \end{aligned}$$

The nominal joint configuration was set to $\boldsymbol{\theta }_0\!=\![\pi /4, \pi /6, \pi /8]^T$, yielding a target end-effector position $\textbf{p}_0\!=\![1.948, 1.330]^T$. This configuration was chosen to avoid singularities and to position the arm in a mid-range posture representative of static pointing tasks.

### Notation: Across-trial versus within-trial computations

Throughout this manuscript, computational steps fall into two distinct categories that we label explicitly. *Within-trial* operations act on a single trial *i* across the within-trial time index $t\!\in \!\{1, \ldots , N_t\}$; examples include the joint-angle trajectory $\boldsymbol{\theta }^{(i)}(t)$, the projected single-trial deviation series $u^{(i)}(t)\!=\!\textbf{n}^{T}\Delta \boldsymbol{\theta }^{(i)}(t)$, and any temporal statistic (autocorrelation, DFA, spectral slope) computed on such a series. *Across-trial* operations aggregate over the trial index $i\!\in \!\{1, \ldots , N_{\text {trials}}\}$ at a fixed within-trial time point; examples include the standard UCM variance components $V_{\text {UCM}}(t)$ and $V_{\text {ORT}}(t)$, which are variances computed across trials at each instant *t*, and the time-resolved ratio $r_{\text {UCM}}(t)$. A third class—*within-trial computations followed by across-trial inferential testing*—forms the basis of the per-trial analyses: a temporal statistic is computed within each trial separately, yielding a distribution of $N_{\text {trials}}$ values per condition that is then submitted to non-parametric testing across conditions. The empirical motivation for this third class is that the projection operation preserves temporal structure within trials, but the across-trial aggregation step that produces $V_{\text {UCM}}(t)$ discards it. Where ambiguity might arise in subsequent sections, we annotate equations and procedures explicitly with “(within-trial)” or “(across-trial)” to make the operation clear.

### Jacobian and subspace decomposition

The Jacobian matrix $\textbf{J}\!\in \!\mathbb {R}^{2 \times 3}$ relating joint velocities to end-effector velocities was computed analytically:

5 $$\begin{aligned} \textbf{J} = \frac{\partial \textbf{p}}{\partial \boldsymbol{\theta }} = \begin{bmatrix} -L_1 s_1 - L_2 s_{12} - L_3 s_{123} & -L_2 s_{12} - L_3 s_{123} & -L_3 s_{123} \\ L_1 c_1 + L_2 c_{12} + L_3 c_{123} & L_2 c_{12} + L_3 c_{123} & L_3 c_{123} \end{bmatrix}, \end{aligned}$$

where $s_i\!=\!\sin (\theta _i)$, $c_i\!=\!\cos (\theta _i)$, $s_{12}\!=\!\sin (\theta _1\!+\!\theta _2)$, $c_{12}\!=\!\cos (\theta _1\!+\!\theta _2)$, and similarly for $s_{123}$ and $c_{123}$.

At the nominal configuration $\boldsymbol{\theta }_0$, the Jacobian was evaluated and decomposed via singular value decomposition (SVD):

6 $$\begin{aligned} \textbf{J} = \textbf{U} \boldsymbol{\Sigma } \textbf{V}^T, \end{aligned}$$

where $\textbf{U}\!\in \!\mathbb {R}^{2 \times 2}$, $\boldsymbol{\Sigma }\!\in \!\mathbb {R}^{2 \times 3}$, and $\textbf{V}\!\in \!\mathbb {R}^{3 \times 3}$. The null space (UCM) was defined by the third column of $\textbf{V}$, denoted $\textbf{n}\!=\!\textbf{V}_{:,3}$, satisfying $\textbf{J}\textbf{n}\!=\!\textbf{0}$ to machine precision. The range space (orthogonal subspace) was spanned by the first two columns $\textbf{R}\!=\!\textbf{V}_{:,1:2}$. Projection operators were constructed as:

7 $$\begin{aligned} \textbf{P}_{\text {UCM}}&= \textbf{n}\textbf{n}^T, \end{aligned}$$

8 $$\begin{aligned} \textbf{P}_{\text {ORT}}&= \textbf{R}\textbf{R}^T = \textbf{I}_3 - \textbf{n}\textbf{n}^T, \end{aligned}$$

where $\textbf{I}_3$ is the $3\!\times \!3$ identity matrix.

### Generation of temporal variability structures

Three distinct temporal structures were imposed on multi-joint trajectories over a *10*-second interval, discretized into $N_t\!=\!100$ time points. For each condition, $N_{\text {trials}}\!=\!200$ trials were generated. Variability was introduced in both task space and joint space to model realistic coordinated motor control.

#### Isochronous (periodic) variability

Isochronous variability was modeled with a deterministic periodic structure in task space at frequency $f_0\!=\!0.1$ Hz; the within-UCM contribution shared this frequency but carried a trial- and time-varying stochastic amplitude (Eq. 11). For each trial *i*, task-space perturbations followed circular trajectories:

9 $$\begin{aligned} \delta p_x^{(i)}(t)&= A_{\text {task}} \cos (2\pi f_0 t + \phi _{\text {task}}^{(i)}), \end{aligned}$$

10 $$\begin{aligned} \delta p_y^{(i)}(t)&= A_{\text {task}} \sin (2\pi f_0 t + \phi _{\text {task}}^{(i)}), \end{aligned}$$

where $A_{\text {task}}\!=\!0.02$ and $\phi _{\text {task}}^{(i)}\!\sim \!\mathcal {U}(0, 2\pi )$ was a random phase. These perturbations were mapped to joint space via the pseudoinverse:

11 $$\begin{aligned} \delta \boldsymbol{\theta }_{\text {task}}^{(i)}(t) = \textbf{J}^{\dagger } \boldsymbol{\delta p}^{(i)}(t), \end{aligned}$$

where $\textbf{J}^{\dagger }\!=\!\textbf{V}\boldsymbol{\Sigma }^{\dagger }\textbf{U}^T$ is the Moore–Penrose pseudoinverse, with $\boldsymbol{\Sigma }^{\dagger }$ denoting the pseudoinverse of the rectangular diagonal matrix $\boldsymbol{\Sigma }\in \mathbb {R}^{2\times 3}$, formed by transposing and taking the reciprocal of the non-zero singular values.

Additional variability within the UCM was introduced as:

12 $$\begin{aligned} \delta \boldsymbol{\theta }_{\text {UCM}}^{(i)}(t) = A_{\text {UCM}}^{(i)}(t)\, \sin \!\big (2\pi f_0 t + \phi _{\text {UCM}}^{(i)}\big )\, \textbf{n}, \end{aligned}$$

where the phase $\phi _{\text {UCM}}^{(i)}\!\sim \!\mathcal {U}(0, 2\pi )$ was drawn once per trial and the amplitude $A_{\text {UCM}}^{(i)}(t)\!\sim \!\mathcal {U}(0, 0.1)$ was resampled independently at each time point. The UCM-space contribution in the isochronous condition is therefore a periodic carrier at $f_0$ with a stochastically modulated amplitude, while the task-space contribution (Eq. 9) remains a deterministic circular oscillation. The total joint configuration was:

13 $$\begin{aligned} \boldsymbol{\theta }^{(i)}(t) = \boldsymbol{\theta }_0 + \delta \boldsymbol{\theta }_{\text {task}}^{(i)}(t) + \delta \boldsymbol{\theta }_{\text {UCM}}^{(i)}(t). \end{aligned}$$

#### White noise variability

White noise variability was generated as temporally uncorrelated Gaussian perturbations. At each time point *t* and trial *i*, independent samples were drawn:

14 $$\begin{aligned} \boldsymbol{\delta p}^{(i)}(t)&\sim \mathcal {N}(\textbf{0}, \sigma _{\text {task}}^2 \textbf{I}_2), \quad \sigma _{\text {task}} = 0.02, \end{aligned}$$

15 $$\begin{aligned} \delta \boldsymbol{\theta }_{\text {task}}^{(i)}(t)&= \textbf{J}^{\dagger } \boldsymbol{\delta p}^{(i)}(t), \end{aligned}$$

16 $$\begin{aligned} \delta \theta _{\text {UCM}}^{(i)}(t)&\sim \mathcal {N}(0, \sigma _{\text {UCM}}^2), \quad \sigma _{\text {UCM}} = 0.1, \end{aligned}$$

17 $$\begin{aligned} \delta \boldsymbol{\theta }_{\text {UCM}}^{(i)}(t)&= \delta \theta _{\text {UCM}}^{(i)}(t) \textbf{n}. \end{aligned}$$

The joint configuration was constructed in the same way as in Eq. 13.

#### Pink noise variability

Pink noise exhibits power spectral density $S(f)\!\propto \!1/f$, characteristic of long-range temporal correlations. Pink noise time series were generated using the Voss–McCartney algorithm (Voss and Clarke 1978), which superposes multiple binary noise sources updated at different rates. For a time series of length $N_t$, $M\!=\!\lceil \log _2(N_t) \rceil$ independent Gaussian sources were maintained. At each time step *k*, sources corresponding to binary digits in the representation of *k* were resampled, and the pink noise sample was the sum of all sources. This procedure was applied independently to generate:

1. Two task-space pink noise signals for $\delta p_x(t)$ and $\delta p_y(t)$,
2. One joint-space pink noise signal for the UCM component.

Each pink noise signal was normalized to zero mean and standard deviation matching the white noise condition ($\sigma _{\text {task}}\!=\!0.02$ for task space, $\sigma _{\text {UCM}}\!=\!0.1$ for UCM space). The mapping to joint space proceeded as in the white noise case.

### Uncontrolled manifold analysis

For each condition and time point *t*, joint angle deviations from the mean time series were computed:

18 $$\begin{aligned} \Delta \boldsymbol{\theta }^{(i)}(t)&= \boldsymbol{\theta }^{(i)}(t) - \bar{\boldsymbol{\theta }}(t), \end{aligned}$$

19 $$\begin{aligned} \bar{\boldsymbol{\theta }}(t)&= \frac{1}{N_{\text {trials}}} \sum _{i=1}^{N_{\text {trials}}} \boldsymbol{\theta }^{(i)}(t). \end{aligned}$$

Variance components were calculated per standard UCM methodology:

20 $$\begin{aligned} V_{\text {UCM}}(t)&= \frac{1}{N_{\text {trials}} \cdot \dim (\mathcal {N})} \sum _{i=1}^{N_{\text {trials}}} \Vert \textbf{P}_{\text {UCM}} \Delta \boldsymbol{\theta }^{(i)}(t) \Vert ^2 \end{aligned}$$

21 $$\begin{aligned}&= \frac{1}{N_{\text {trials}} \cdot 1} \sum _{i=1}^{N_{\text {trials}}} (\textbf{n}^T \Delta \boldsymbol{\theta }^{(i)}(t))^2, \end{aligned}$$

22 $$\begin{aligned} V_{\text {ORT}}(t)&= \frac{1}{N_{\text {trials}} \cdot \dim (\mathcal {R})} \sum _{i=1}^{N_{\text {trials}}} \Vert \textbf{P}_{\text {ORT}} \Delta \boldsymbol{\theta }^{(i)}(t) \Vert ^2 \end{aligned}$$

23 $$\begin{aligned}&= \frac{1}{N_{\text {trials}} \cdot 2} \sum _{i=1}^{N_{\text {trials}}} \Vert \textbf{R}^T \Delta \boldsymbol{\theta }^{(i)}(t) \Vert ^2, \end{aligned}$$

and the UCM variance ratio was computed as:

24 $$\begin{aligned} r_{\text {UCM}}(t) = \frac{V_{\text {UCM}}(t)}{V_{\text {ORT}}(t)}. \end{aligned}$$

Summary statistics were obtained by averaging over time. Consistent with standard UCM practice, which averages the ratio (or the synergy index) across observations rather than forming a ratio of averages, we report the time-average of the instantaneous ratio,

25 $$\begin{aligned}&\bar{V}_{\text {UCM}} = \frac{1}{N_t} \sum _{t=1}^{N_t} V_{\text {UCM}}(t), \quad \bar{V}_{\text {ORT}} = \frac{1}{N_t} \sum _{t=1}^{N_t} V_{\text {ORT}}(t),\nonumber \\ &\quad \bar{r}_{\text {UCM}} = \frac{1}{N_t}\sum _{t=1}^{N_t} r_{\text {UCM}}(t) = \frac{1}{N_t}\sum _{t=1}^{N_t}\frac{V_{\text {UCM}}(t)}{V_{\text {ORT}}(t)}. \end{aligned}$$

The ratio of the time-averaged components, $\bar{V}_{\text {UCM}}/\bar{V}_{\text {ORT}}$, differs from this quantity by $\approx \!1\%$ in every condition; where both are quoted we distinguish them explicitly. Neither summary preserves temporal ordering, which is the property at issue here.

### Temporal structure characterization

#### Autocorrelation function

The autocorrelation function (ACF) quantifies temporal dependencies in the variance time series (Box et al. 2015; Chatfield and Xing 2019). For a time series *x(t)* with mean *μ * and variance $\sigma ^2$, the ACF at lag *τ * is:

26 $$\begin{aligned} \rho (\tau ) = \frac{1}{\sigma ^2(N_t - \tau )} \sum _{t=1}^{N_t - \tau } (x(t) - \mu )(x(t+\tau ) - \mu ). \end{aligned}$$

We computed ACF for both $V_{\text {UCM}}(t)$ and $V_{\text {ORT}}(t)$ up to maximum lag $\tau _{\max }\!=\!10$ time points. The lag-1 autocorrelation *ρ (1)* served as a primary index of temporal correlation: $\rho (1)\!\approx \!0$ indicates white noise, $\rho (1)\!>\!0.5$ indicates positive temporal correlation, and $\rho (1)\!\approx \!1$ indicates strong persistence.

#### Power spectral density

Power spectral density (PSD) was estimated using Welch’s 1967 method implemented via MATLAB’s pwelch function. The variance time series was divided into overlapping segments of length *32* with *50%* overlap (16 samples), each windowed with a Hamming function. The periodogram of each segment was computed via discrete Fourier transform with nfft = 256, and PSDs were averaged across segments:

27 $$\begin{aligned} \hat{S}(f) = \frac{1}{K} \sum _{k=1}^{K} \left| \text {DFT}\left( w(t) x_k(t) \right) \right| ^2, \end{aligned}$$

where *K* is the number of segments, *w(t)* is the Hamming window function, and $x_k(t)$ is the *k*-th segment. Sampling frequency was set to $f_s\!=\!10$ Hz.

Spectral slopes were characterized by linear regression in log–log space over the frequency range $0.01\!<\!f\!<\!1$ Hz:

28 $$\begin{aligned} \log _{10} \hat{S}(f) = \beta \log _{10}(f) + c, \end{aligned}$$

where *β * is the spectral exponent. White noise exhibits $\beta \!\approx \!0$ (flat spectrum), pink noise exhibits $\beta \!\approx \!-1$, and periodic signals exhibit peaks at characteristic frequencies.

#### Detrended fluctuation analysis

Detrended fluctuation analysis (DFA) quantifies long-range correlations robust to nonstationarities (Peng et al. 1994). The cumulative sum of the mean-centered time series was computed:

29 $$\begin{aligned} Y(k) = \sum _{t=1}^{k} \left( x(t) - \bar{x} \right) . \end{aligned}$$

The cumulative series was divided into non-overlapping windows of size *n*, and a linear trend $Y_n(k)$ (first-order polynomial) was fit within each window via least squares. The fluctuation function was:

30 $$\begin{aligned} F(n) = \sqrt{ \frac{1}{N_t} \sum _{k=1}^{N_t} \left[ Y(k) - Y_n(k) \right] ^2 }. \end{aligned}$$

This procedure was repeated for logarithmically spaced window sizes $4\!\le \!n\!\le \!N_t/4$, with 20 scales uniformly distributed in logarithmic space. The scaling exponent *α * was obtained from linear regression in log–log space:

31 $$\begin{aligned} \log _{10} F(n) = \alpha \log _{10}(n) + c. \end{aligned}$$

The exponent *α * characterizes temporal correlations: $\alpha \!\approx \!0.5$ indicates uncorrelated noise (white), $\alpha \!\approx \!1.0$ indicates *1/f* noise (pink), and $\alpha \!>\!1.0$ indicates nonstationary persistent correlations.

### End-effector variance

To verify task-space performance, end-effector position variance was computed at each time point:

32 $$\begin{aligned} V_{\text {EE}}(t) = \frac{1}{2N_{\text {trials}}} \sum _{i=1}^{N_{\text {trials}}} \Vert \textbf{p}^{(i)}(t) - \bar{\textbf{p}}(t) \Vert ^2, \end{aligned}$$

where $\textbf{p}^{(i)}(t)\!=\![p_x(\boldsymbol{\theta }^{(i)}(t)), p_y(\boldsymbol{\theta }^{(i)}(t))]^T$ and the factor of two accounts for dimensionality. End-effector variance depends on the UCM decomposition: to first order, it is the task-space image of the ORT (range-space) variance, because $V_{\text {ORT}}$ collects precisely the joint-angle variance that maps, through $\textbf{J}$, onto changes in $\textbf{p}$. We therefore use $\bar{V}_{\text {EE}}$ as a direct task-space check that the imposed variability produced the intended performance dispersion, complementing the joint-space partition.

### Variance ratio time series analysis

To examine whether temporal structure manifests in the time evolution of UCM variance ratios, we computed the instantaneous ratio time series $r_{\text {UCM}}(t)$ and applied temporal characterization methods (autocorrelation, power spectral density). We also asked whether the ratio could inherit the temporal structure of its constituents. This depends on how numerator and denominator co-vary: if $V_{\text {UCM}}(t)$ and $V_{\text {ORT}}(t)$ fluctuate independently, then their pointwise ratio mixes two unrelated series and need not preserve the autocorrelation or scaling of either. We therefore quantified the coupling between the two components as the zero-lag cross-correlation, computed as the Pearson correlation:

33 $$\begin{aligned} \rho _{\text {cross}} = \frac{\sum _{t=1}^{N_t} (V_{\text {UCM}}(t) - \bar{V}_{\text {UCM}})(V_{\text {ORT}}(t) - \bar{V}_{\text {ORT}})}{\sqrt{\sum _{t=1}^{N_t} (V_{\text {UCM}}(t) - \bar{V}_{\text {UCM}})^2} \sqrt{\sum _{t=1}^{N_t} (V_{\text {ORT}}(t) - \bar{V}_{\text {ORT}})^2}}. \end{aligned}$$

### Per-trial geometric and temporal indices

To enable inferential comparisons across conditions, we computed per-trial values of all dependent variables. For each trial *i*, the deviation series $\Delta \boldsymbol{\theta }^{(i)}(t)\!=\!\boldsymbol{\theta }^{(i)}(t)\!-\!\bar{\boldsymbol{\theta }}(t)$ was projected onto the UCM and ORT subspaces, yielding a one-dimensional time series $u^{(i)}(t)\!=\!\textbf{n}^{T}\Delta \boldsymbol{\theta }^{(i)}(t)$ and a two-dimensional time series $\textbf{o}^{(i)}(t)\!=\!\textbf{R}^{T}\Delta \boldsymbol{\theta }^{(i)}(t)$. This projection (a constant linear operator) preserves within-trial temporal structure; the cross-trial aggregation step of standard UCM is what discards it. Per-trial geometric indices were:

34 $$\begin{aligned} V_{\text {UCM}}^{(i)}&= \textrm{Var}_{t}\!\left[ u^{(i)}(t)\right] , \end{aligned}$$

35 $$\begin{aligned} V_{\text {ORT}}^{(i)}&= \tfrac{1}{2}\sum _{j=1}^{2}\textrm{Var}_{t}\!\left[ o_j^{(i)}(t)\right] , \end{aligned}$$

36 $$\begin{aligned} r_{\text {UCM}}^{(i)}&= V_{\text {UCM}}^{(i)} / V_{\text {ORT}}^{(i)}. \end{aligned}$$

The normalized variance difference was computed with dimensional normalization, following the synergy-index construction of Latash Latash (2010):

37 $$\begin{aligned} \Delta V^{(i)} = \frac{V_{\text {UCM}}^{(i)}/\dim (\mathcal {N}) - V_{\text {ORT}}^{(i)}/\dim (\mathcal {R})}{\left( V_{\text {UCM}}^{(i)} + V_{\text {ORT}}^{(i)}\right) /(\dim (\mathcal {N})+\dim (\mathcal {R}))}. \end{aligned}$$

For our system $\dim (\mathcal {N})\!=\!1$ and $\dim (\mathcal {R})\!=\!2$, *Δ V* is confined to the *asymmetric* interval $[\Delta V_{\min }, \Delta V_{\max }]$, with

38 $$\begin{aligned} \Delta V_{\max } = \frac{\dim (\mathcal {N})+\dim (\mathcal {R})}{\dim (\mathcal {N})} = 3 \;\; (\text {attained when } V_{\text {ORT}}\!=\!0), \end{aligned}$$

39 $$\begin{aligned} \Delta V_{\min } = -\,\frac{\dim (\mathcal {N})+\dim (\mathcal {R})}{\dim (\mathcal {R})} = -\tfrac{3}{2} \;\; (\text {attained when } V_{\text {UCM}}\!=\!0). \end{aligned}$$

Because the interval is asymmetric, the variance-stabilizing *Z*-transform must reference *both* bounds. The general logit-type transform for a variable confined to $[\Delta V_{\min }, \Delta V_{\max }]$ is

40 $$\begin{aligned} \Delta V_z^{(i)} = \tfrac{1}{2} \ln \!\left( \frac{\Delta V^{(i)} - \Delta V_{\min }}{\Delta V_{\max } - \Delta V^{(i)}} \right) , \end{aligned}$$

which maps $(\Delta V_{\min }, \Delta V_{\max })$ onto $(-\infty , +\infty )$ and reduces to the familiar symmetric Fisher form $\tfrac{1}{2}\ln \!\big [(1+\Delta V/\Delta V_{\max })/(1-\Delta V/\Delta V_{\max })\big ]$ only when the bounds are treated as symmetric $\pm \Delta V_{\max }$. We note that neither closed form is given explicitly by Latash (2010), whom we cite for the index *Δ V* itself; the *Z*-transform is our variance-stabilizing choice. Crucially, every inferential statistic reported below is rank-based (Kruskal–Wallis *H*, Mann–Whitney *U*, Cliff’s *δ *) and therefore invariant under any strictly monotonic transformation of *Δ V*; the choice among these transforms leaves every effect size and *p*-value in Table 1 unchanged and affects only the numerical scale of the descriptive $\Delta V_z$ means. The descriptive $\Delta V_z$ means in Table 1 were computed with the symmetric special case; adopting Eq. 40 rescales those means but, being monotonic, alters none of the reported statistics. Per-trial temporal indices—DFA scaling exponent *α *, lag-1 autocorrelation *ρ (1)*, and spectral slope *β *—were computed on $u^{(i)}(t)$ for the UCM component and on the magnitude series $\Vert \textbf{o}^{(i)}(t)\Vert$ for the ORT component, yielding per-condition distributions of $N_{\text {trials}}\!=\!200$.

### Statistical analysis

Shapiro–Wilk tests rejected normality for several variables ($p\!<\!0.05$), and Levene’s tests indicated heterogeneity of variance, motivating non-parametric inference throughout. We used Kruskal–Wallis *H* tests for omnibus comparisons across the three conditions, with effect size reported as $\eta ^2_H\!=\!(H\!-\!k\!+\!1)/(N\!-\!k)$ ($k\!=\!3$, $N\!=\!600$). Pairwise comparisons used two-sided Mann–Whitney *U* tests with Bonferroni correction for three contrasts ($\alpha _{\text {adj}}\!=\!0.05/3\!=\!0.0167$). Effect sizes were quantified using Cliff’s *δ *, interpreted as negligible ($|\delta |\!<\!0.147$), small ($0.147\!\le \!|\delta |\!<\!0.330$), medium ($0.330\!\le \!|\delta |\!<\!0.474$), or large ($|\delta |\!\ge \!0.474$).

We emphasize that with $N_{\text {trials}}\!=\!200$ per condition, even trivial differences may achieve statistical significance. Our interpretation, therefore, centers on effect sizes: a Bonferroni-significant but small effect ($|\delta |\!<\!0.147$) is treated as practical equivalence, while a large effect ($|\delta |\!\ge \!0.474$) constitutes meaningful separation. All analyses were implemented in MATLAB R2024a (Mathworks, Natick, MA).

## Results

We applied UCM analysis to simulated reaching with three temporal structures—isochronous (periodic), white noise (uncorrelated), and pink noise (*1/f*)—at an identical nominal configuration and task geometry. Despite order-of-magnitude differences in temporal dynamics, the time-averaged variance ratios were similar for white and pink noise, showing that the time-averaged summary is insensitive to temporal correlation.

### Joint-space and task-space kinematics differ across conditions while sharing the nominal configuration

**Fig. 3:**
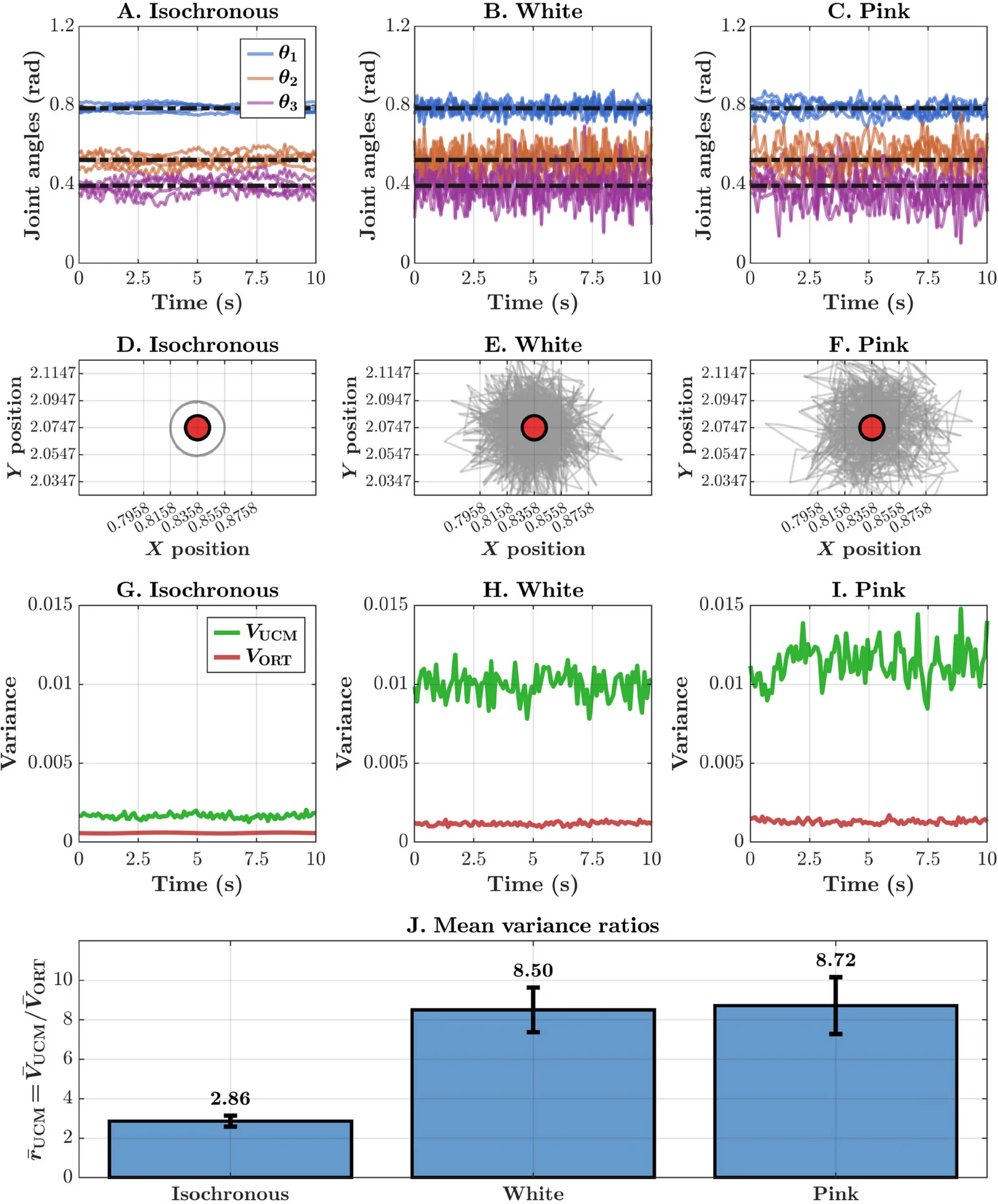
The time-averaged UCM variance ratio is invariant to temporal correlation structure in stochastic variability, even though the time-resolved components are not. (**A–C**) Joint angle trajectories for five representative trials across three conditions: (**A**) isochronous (deterministic periodic oscillations at *0.1* Hz with random phase across trials), (**B**) white noise (temporally uncorrelated Gaussian fluctuations), (**C**) pink noise (*1/f* power spectral density with long-range temporal correlations). Blue, orange, and purple lines represent joints $\theta _1$, $\theta _2$, and $\theta _3$ respectively. Dashed black lines indicate the nominal joint configuration, which is identical across conditions. (**D–F**) End-effector trajectories in Cartesian space for ten trials. Red circle indicates the target position. (**D**) Isochronous condition produces deterministic circular task-space oscillations with tight spatial clustering due to coherent motion in the range space of the Jacobian. (**E**) White noise produces irregular, spatially dispersed fluctuations with no temporal correlations. (**F**) Pink noise produces smoother trajectories with persistent deviations due to long-range temporal correlations. (**G–I**) Time series of UCM variance (green) and ORT variance (red) across *100* time points. The isochronous condition (**G**) exhibits low variance in both subspaces due to deterministic structure. White noise (**H**) and pink noise (**I**) show higher variance magnitudes that remain similar despite fundamentally different temporal correlation properties. (**J**) Mean variance ratio $\bar{r}_{\text {UCM}}\!=\!\bar{V}_{\text {UCM}}/\bar{V}_{\text {ORT}}$ averaged over time. The isochronous condition (*2.86*) differs from stochastic conditions because deterministic circular motion in task space geometrically inflates range-space variance, a purely spatial effect. White noise (*8.50*) and pink noise (*8.72*) produce similar ratios (difference *2.6%*) despite order-of-magnitude differences in temporal correlation structure (white: $\rho (1) \approx 0$; pink: $\rho (1)\!=\!0.25$), demonstrating UCM insensitivity to temporal dependencies in stochastic variability. Error bars represent standard deviation across time points

Joint angle trajectories exhibited qualitatively distinct coordination patterns across conditions (Fig. 3A–C). Isochronous variability produced smooth, periodic oscillations at *0.1* Hz, with trial-to-trial phase variability but a consistent frequency, reflecting deterministic temporal structure (Fig. 3A). White noise generated irregular, uncorrelated fluctuations with no discernible temporal pattern, consistent with memoryless stochastic variability (Fig. 3B). Pink noise produced persistent deviations that evolved smoothly across time, characteristic of long-range temporal correlations and *1/f* dynamics (Fig. 3C). All three conditions maintained the same nominal joint configuration (dashed black lines), differing only in temporal correlation structure rather than geometric configuration.

End-effector trajectories reflected these differences in task-space behavior (Fig. 3D–F). Isochronous variability produced circular task-space oscillations with tight spatial clustering around the target (Fig. 3D). White noise generated irregular, spatially dispersed fluctuations (Fig. 3E). Pink noise also yielded spatially dispersed but smoother trajectories with persistent deviations due to long-range temporal correlations (Fig. 3F). Mean end-effector variance was $\bar{V}_{\text {EE}}\!=\!2.01\!\times \!10^{-5}$ for isochronous, $4.03\!\times \!10^{-5}$ for white noise, and $4.67\!\times \!10^{-5}$ for pink noise. The lower variance in the isochronous condition reflects deterministic circular trajectories that maintained tighter spatial clustering.

### Time-averaged variance ratios are equal for white and pink noise

Despite these qualitative differences in joint-space and task-space dynamics, UCM variance decomposition produced similar partitions for white noise and pink noise (Fig. 3G–J). Variance time series for both $V_{\text {UCM}}(t)$ (green) and $V_{\text {ORT}}(t)$ (red) exhibited similar magnitudes across conditions (Fig. 3G–I), with no systematic differences distinguishing white from pink noise at the level of instantaneous variance geometry.

Mean UCM variance ratios were $\bar{r}_{\text {UCM}}\!=\!2.86$ for isochronous, *8.50* for white noise, and *8.72* for pink noise (Fig. 3J). The absolute difference between white noise and pink noise was *0.22*, representing *2.6%* relative difference. Time-averaged variance components revealed similarly close agreement: for white noise, $\bar{V}_{\text {UCM}}\!=\!1.13\!\times \!10^{-2}$ and $\bar{V}_{\text {ORT}}\!=\!1.30\!\times \!10^{-3}$; for pink noise, $\bar{V}_{\text {UCM}}\!=\!1.15\!\times \!10^{-2}$ and $\bar{V}_{\text {ORT}}\!=\!1.30\!\times \!10^{-3}$, differing by less than *2%* for both components, despite different temporal ordering and dependence across time.

The lower isochronous ratio (*2.86*) reflects the imposed deterministic structure through a purely spatial mechanism: coherent circular task-space oscillation geometrically inflates variance in the range space of the Jacobian, raising $V_{\text {ORT}}$ relative to $V_{\text {UCM}}$. The critical comparison is white versus pink noise, which differ fundamentally in autocorrelation (white: $\rho (\tau )\!\approx \!0$; pink: slowly decaying, *1/f*) yet yield indistinguishable time-averaged ratios.

The reason is worth stating explicitly. The across-trial components $V_{\text {UCM}}(t)$ and $V_{\text {ORT}}(t)$ pool, at each fixed instant *t*, the projected deviations of $N_{\text {trials}}$ independent trials. Pink structure is a *within-trial* property—how a trial’s deviation at *t* relates to its own deviation at $t\!+\!1$, $t\!+\!2$, and so on. Sampling one instant across independent trials severs those links: at fixed *t*, the pink trials are mutually independent draws with the same marginal spread as the white condition, so the instantaneous cross-trial distribution is white-like in both. Identical $\bar{V}_{\text {UCM}}$ and $\bar{V}_{\text {ORT}}$ follow as a direct consequence. The collapse is localized to this across-trial-at-fixed-*t* step, and each trial’s projected series still carries its within-trial signature. The time-averaged ratio resolves coarse spatial differences—separating isochronous (*2.86*) from both stochastic conditions ($\approx \!8.5$)—while assigning matched values to two stochastic processes that share instantaneous covariance.

### Autocorrelation of the variance components differs across white, pink, and isochronous conditions

**Fig. 4:**
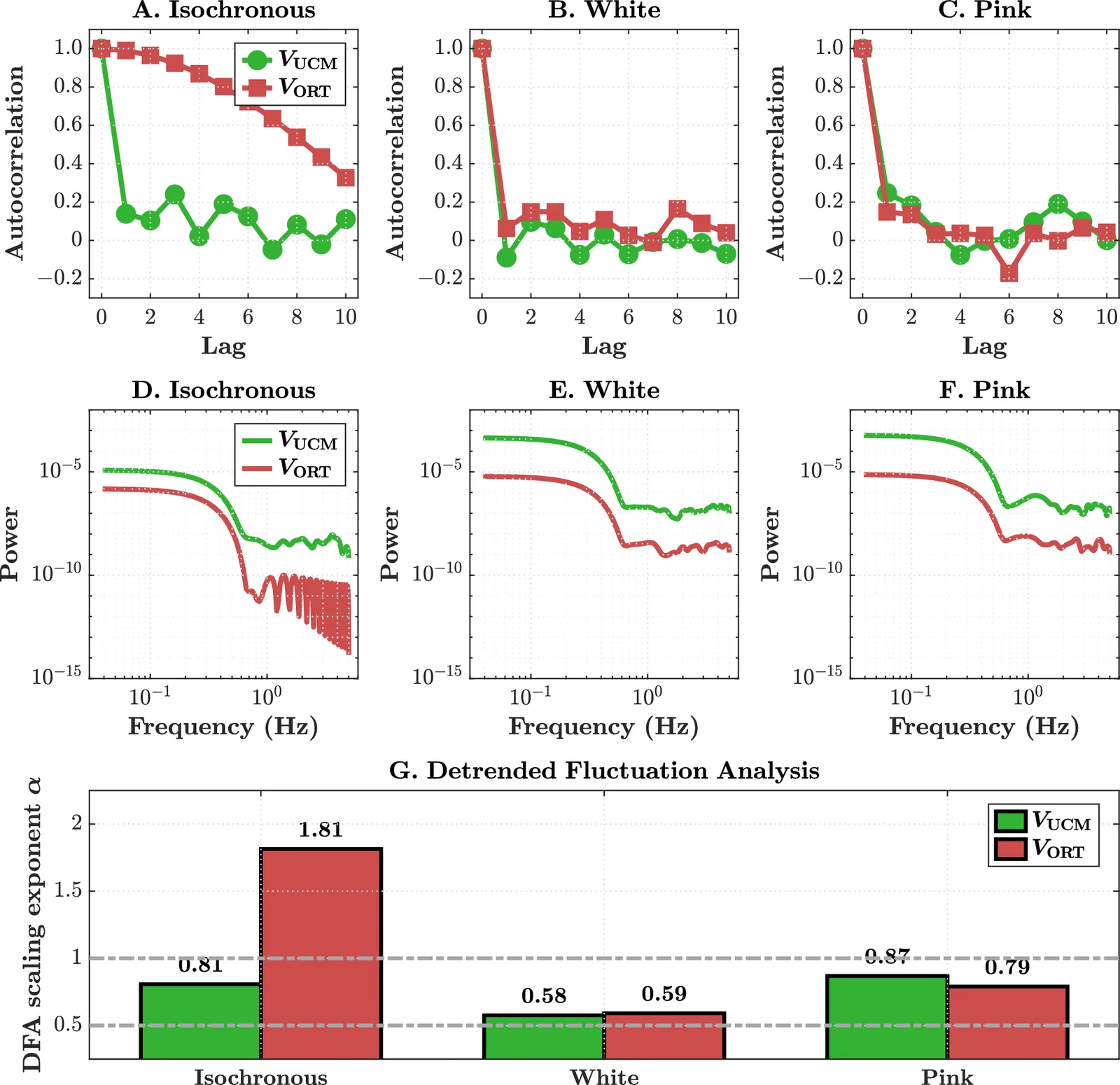
Temporal structure analysis reveals differences invisible to UCM decomposition. (**A–C**) Autocorrelation functions (ACF) for $V_{\text {UCM}}(t)$ (green) and $V_{\text {ORT}}(t)$ (red) up to lag *10*. (**A**) Isochronous: High autocorrelation in $V_{\text {ORT}}$ ($\rho (1)\!=\!0.98$) reflects periodic task-space oscillations; low autocorrelation in $V_{\text {UCM}}$ ($\rho (1)\!=\!0.14$) reflects trial-to-trial phase variability. (**B**) White noise: Near-zero autocorrelation for both components ($\rho _{\text {UCM}}(1)\!=\!-0.09$, $\rho _{\text {ORT}}(1)\!=\!0.10$), consistent with temporally uncorrelated processes. (**C**) Pink noise: Low positive autocorrelation ($\rho _{\text {UCM}}(1)\!=\!0.25$, $\rho _{\text {ORT}}(1)\!=\!0.17$), reflecting *1/f* temporal structure with slowly decaying temporal correlations. (**D–F**) Power spectral density (PSD) estimated via Welch’s method, plotted in log–log coordinates. (**D**) Isochronous: Clear spectral peak at *0.1* Hz for both UCM and ORT components. (**E**) White noise: Broadband spectrum ($\beta _{\text {UCM}}\!=\!-3.43$) indicating distributed power across frequencies. (**F**) Pink noise: Broadband spectrum ($\beta _{\text {UCM}}\!=\!-3.32$) with similar scaling to white noise in this analysis window. Spectral slopes differ minimally ($\Delta \beta \!=\!0.11$), indicating both conditions exhibit similar frequency characteristics despite distinct temporal correlation structures revealed by ACF and DFA. (**G**) Detrended fluctuation analysis (DFA) scaling exponents. White noise ($\alpha _{\text {UCM}}\!=\!0.58$, $\alpha _{\text {ORT}}\!=\!0.59$) indicates uncorrelated fluctuations; pink noise ($\alpha _{\text {UCM}}\!=\!0.87$, $\alpha _{\text {ORT}}\!=\!0.79$) indicates *1/f* long-range correlations; isochronous ($\alpha _{\text {UCM}}\!=\!0.81$, $\alpha _{\text {ORT}}\!=\!1.81$) shows strong persistent correlations in task-relevant direction. UCM ratios (Fig. 3J) do not reflect these *50%* differences in scaling properties between white noise and pink noise

Autocorrelation functions for $V_{\text {UCM}}(t)$ and $V_{\text {ORT}}(t)$ revealed systematic temporal differences across conditions that are not captured by geometric variance decomposition (Fig. 4A–C). For isochronous variability, $V_{\text {ORT}}$ exhibited high lag-1 autocorrelation ($\rho (1)\!=\!0.98$), reflecting periodic task-space oscillations and strong short-range temporal dependence (Fig. 4A, red squares). In contrast, $V_{\text {UCM}}$ showed low autocorrelation ($\rho (1)\!=\!0.14$), reflecting both trial-to-trial phase variability and the time-varying stochastic amplitude of the UCM-space oscillation, which together weaken temporal alignment across trials (Fig. 4A, green circles).

White noise exhibited near-zero autocorrelation for both components ($\rho _{\text {UCM}}(1)\!=\!-0.09$, $\rho _{\text {ORT}}(1)\!=\!0.10$), consistent with temporally uncorrelated, memoryless processes (Fig. 4B). Pink noise showed low but positive autocorrelation ($\rho _{\text {UCM}}(1)\!=\!0.25$, $\rho _{\text {ORT}}(1)\!=\!0.17$), reflecting slowly decaying correlations characteristic of *1/f* temporal structure (Fig. 4C). The divergence between white noise and pink noise autocorrelation structure—clearly visible across lags in the time-resolved components—was not reflected in the time-averaged variance ratios, which differed by only *2.6%*, underscoring that it is the time-averaged summary, and not the components themselves, that is insensitive to temporal dependence.

### Cross-trial spectral slopes differ little between white and pink noise

Power spectral density (PSD) estimation revealed distinct frequency-domain signatures across conditions (Fig. 4D–F), complementing the autocorrelation results. Isochronous variability exhibited clear spectral peaks at *0.1* Hz for both $V_{\text {UCM}}$ and $V_{\text {ORT}}$, with the peak more pronounced in $V_{\text {ORT}}$ due to coherent phase across trials (Fig. 4D).

White noise produced broadband spectra with estimated spectral slope $\beta _{\text {UCM}}\!=\!-3.43$ (Fig. 4E), while pink noise exhibited similarly broadband spectra with slope $\beta _{\text {UCM}}\!=\!-3.32$ (Fig. 4F). The small difference in slopes ($\Delta \beta \!=\!0.11$) is not, on its own, evidence against temporal dissociation; rather, it reflects two compounding limitations of this estimate. First, Welch-based slope estimation from $N_t\!=\!100$ samples is known to be unreliable: the usable scaling band is narrow, and slope estimates are unstable. Second, and more importantly for our argument, these slopes are computed from the *cross-trial-aggregated* variance time series $V_{\text {UCM}}(t)$ and $V_{\text {ORT}}(t)$—precisely the aggregation step that (Fig. 6) discards within-trial temporal structure. Per-trial PSD slopes computed from single-trial projections, which preserve temporal structure, recover a large white-vs-pink separation (Cliff’s $\delta \!=\!+0.81$, $p\!<\!10^{-44}$; Table 1). The cross-trial PSD result reported here is therefore best understood as a methodological cautionary point: spectral slope is not robust to cross-trial aggregation in short series, and the autocorrelation and DFA analyses below provide the clearer separation.

Despite these frequency-domain similarities at the cross-trial level, time-averaged variance ratios remained similar for white noise and pink noise (*8.50* versus *8.72*); the autocorrelation and DFA results below establish the temporal dissociation more cleanly.

#### Single-trial projections retain the white-versus-pink temporal distinction

**Fig. 5:**
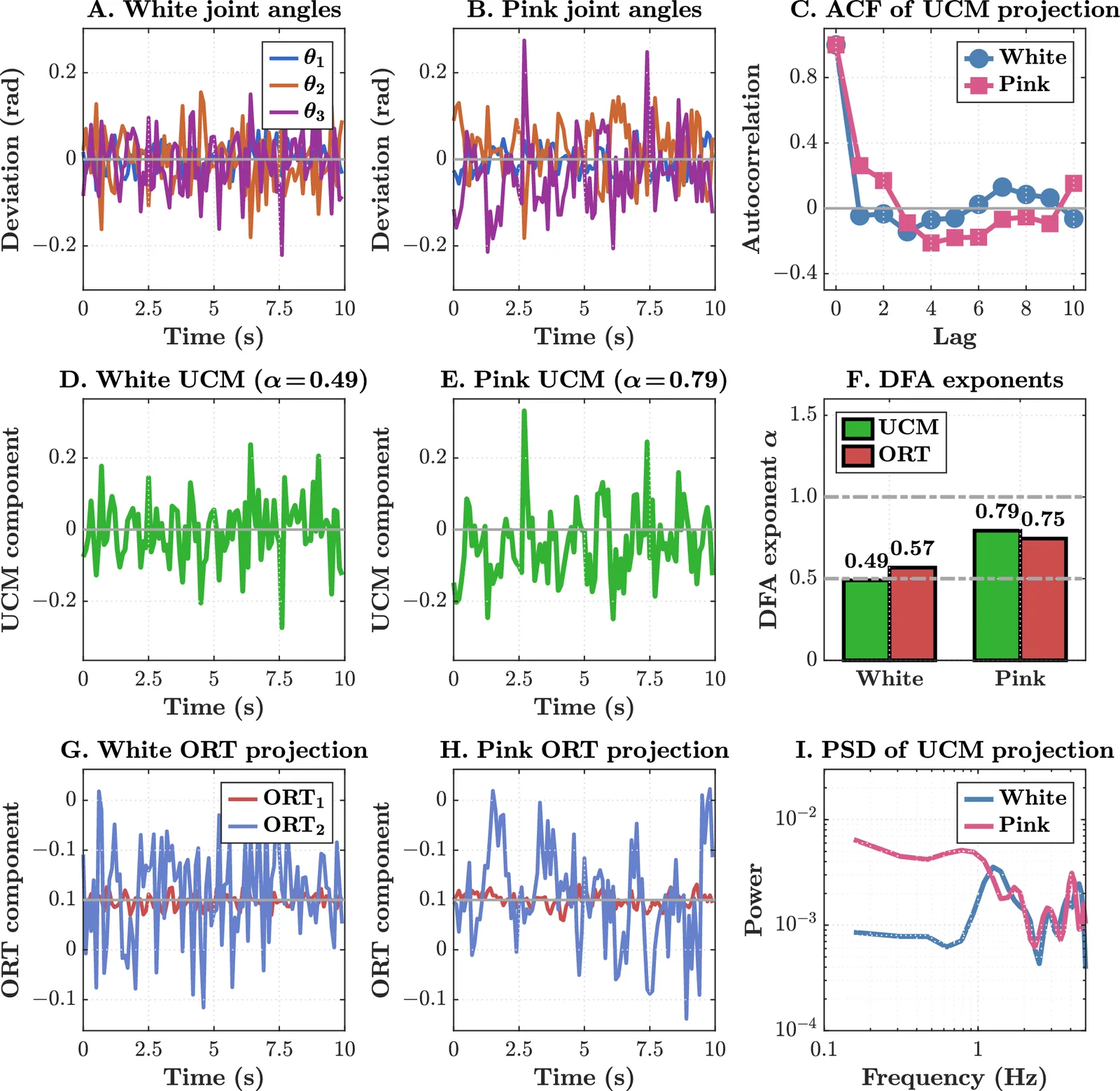
Geometric projection preserves temporal structure; cross-trial aggregation discards it. Single-trial analysis demonstrates that the Jacobian projection operator (a linear transformation) maintains within-trial temporal structure, whereas standard UCM’s cross-trial variance computation discards it. (A–B) Joint angle deviations from the nominal configuration for single trials generated with white-noise (A) and pink-noise (B) temporal structure. (C) Autocorrelation functions of the projected one-dimensional UCM component: white noise exhibits negligible autocorrelation ($\rho (1)\!=\!-0.05$) while pink noise shows positive correlation ($\rho (1)\!=\!0.26$). (D–E) Projection of the white (D) and pink (E) trials onto the one-dimensional UCM subspace. Detrended fluctuation analysis (DFA) yields $\alpha \!=\!0.49$ (uncorrelated) for white noise and $\alpha \!=\!0.79$ (long-range correlations) for pink noise, demonstrating preservation of temporal structure. (F) Summary of DFA scaling exponents, confirming that geometric projection maintains the white-versus-pink separation in both the UCM (*0.49* vs. *0.79*) and ORT (*0.57* vs. *0.75*) components. (G–H) Projection of the white (G) and pink (H) trials onto the two-dimensional ORT subspace, which similarly preserves the temporal distinction. (I) Power spectral density of the projected UCM components, with the pink-noise trial showing greater low-frequency power than the flatter white-noise spectrum. Standard UCM analysis loses this information by computing variance across trials at each time point, aggregating over the within-trial temporal structure that projection preserves

To determine whether temporal information is lost during geometric projection or during cross-trial variance computation, we analyzed single-trial projections onto the UCM and ORT subspaces. We generated individual trials with white-noise (temporally uncorrelated) and pink-noise (long-range correlations with *1/f* scaling) temporal structure (Fig. 5A–B), then projected each trial’s joint angle time series onto the UCM and ORT subspaces defined by the Jacobian.

Detrended fluctuation analysis of the projected time series revealed that the geometric projection preserves temporal structure. For the UCM component (Fig. 5D–E), white noise exhibited $\alpha _{\text {UCM}}\!=\!0.49$ (uncorrelated fluctuations), while pink noise exhibited $\alpha _{\text {UCM}}\!=\!0.79$ (long-range correlations)—a clear separation maintained after projection. Similarly, the first ORT component showed $\alpha _{\text {ORT}}\!=\!0.57$ for white noise and $\alpha _{\text {ORT}}\!=\!0.75$ for pink noise, with both subspaces summarized in Fig. 5F–I. Autocorrelation analysis confirmed this preservation (Fig. 5C): lag-1 autocorrelation for the projected white-noise UCM component was $\rho (1)\!=\!-0.05$, while pink noise showed $\rho (1)\!=\!0.26$.

This demonstrates that the Jacobian projection–a constant linear operator—preserves within-trial variance, so the collapse of that variance occurs in a later, separable step, when the projected data are reduced to a time-averaged variance. The mathematical fact that a time-series statistic can be computed on a projected series leaves a substantive question untouched: what a temporal property of $V_{\text {UCM}}(t)$, $V_{\text {ORT}}(t)$, or a per-trial projection reveals about the control process remains to be established. The projected trajectories are themselves shaped by the geometric operation—the Jacobian, the choice of performance variables, and the null-space decomposition—so their autocorrelation, scaling, or spectral properties reflect that operation as much as any underlying control dynamics. Interpreting these properties as signatures of specific processes, therefore, carries its own inferential burden, one that this study documents rather than discharges. The limitation of the geometric method is that the partition locates variance in task-relevant and task-irrelevant directions, and inferring reading control mechanisms from the temporal behavior of those directions requires independent theoretical and empirical justification.

### DFA scaling exponents separate white from pink noise in the projected components

Detrended fluctuation analysis (DFA) scaling exponents robustly differentiated temporal structures across conditions (Fig. 4G), providing sensitivity to long-range temporal dependence beyond short-lag correlations. White noise yielded $\alpha _{\text {UCM}}\!=\!0.58$ and $\alpha _{\text {ORT}}\!=\!0.59$, consistent with uncorrelated fluctuations ($\alpha \!\approx \!0.5$). Pink noise produced $\alpha _{\text {UCM}}\!=\!0.87$ and $\alpha _{\text {ORT}}\!=\!0.79$, approaching *1/f* scaling indicative of persistent long-range correlations. Isochronous variability showed $\alpha _{\text {UCM}}\!=\!0.81$ and $\alpha _{\text {ORT}}\!=\!1.81$, with the elevated ORT exponent reflecting super-diffusive persistence driven by deterministic periodic task-space dynamics.

The DFA scaling exponents provided independent confirmation of distinct temporal organization. White noise and pink noise differed by $\Delta \alpha \!=\!0.29$ for UCM components (white: *0.58*, pink: *0.87*), representing an approximately *50%* increase in scaling exponent. This pronounced difference in long-range temporal structure contrasts sharply with the *2.6%* difference in time-averaged UCM variance ratios, demonstrating that the time-averaged summary discards information about temporal correlation structure important for mechanistic interpretation—information that the DFA of the projected components measures directly, though its interpretation in control terms remains the open question.

### The instantaneous ratio series shows no white-versus-pink difference

**Fig. 6:**
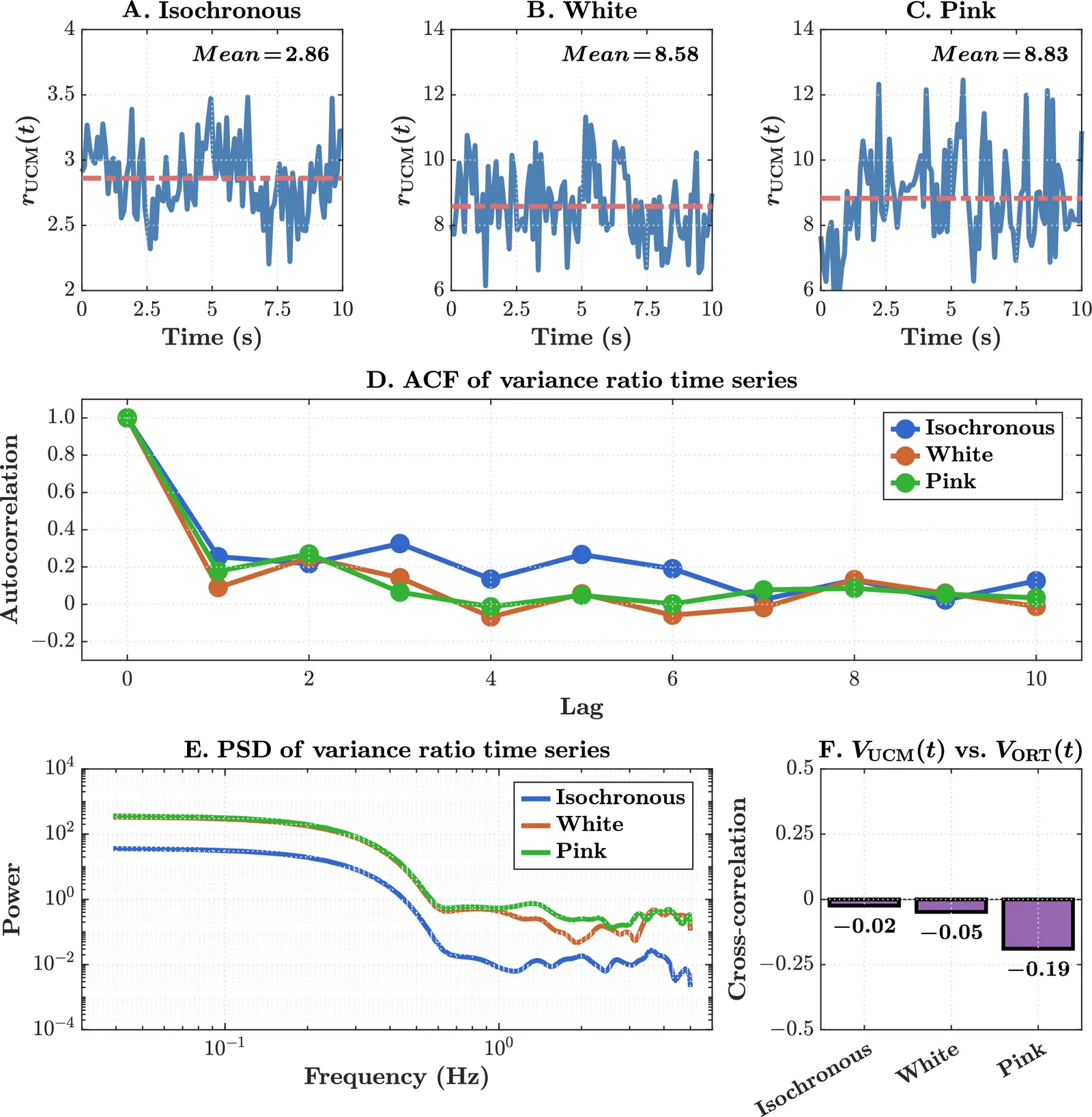
Time-varying UCM variance ratios obscure temporal structure present in constituent components. (**A–C**) Time series of instantaneous variance ratio $r_{\text {UCM}}(t)\!=\!V_{\text {UCM}}(t)/V_{\text {ORT}}(t)$ across *100* time points. (**A**) Isochronous condition exhibits oscillatory fluctuations (mean *2.86*) with period matching the imposed *0.1* Hz frequency, reflecting a phase-dependent relationship between task-space and UCM-space oscillations. (**B**) White noise exhibits stochastic fluctuations around $mean\!=\!8.58$ with no systematic temporal pattern. (**C**) Pink noise exhibits similar stochastic fluctuations around $mean\!=\!8.83$. (**D**) Autocorrelation functions of the variance ratio time series $r_{\text {UCM}}(t)$ for all three conditions. White noise (orange) and pink noise (green) show similar autocorrelation structure despite individual variance components ($V_{\text {UCM}}$ and $V_{\text {ORT}}$) having order-of-magnitude differences in spectral slopes (Fig. 4**D–F**) and long-range correlations (Fig. 4**G**). Isochronous (blue) shows a periodic structure. (**E**) Power spectral density of $r_{\text {UCM}}(t)$. White- and pink-noise ratios exhibit similar spectra, whereas isochronous noise shows a peak at *0.1* Hz corresponding to the imposed oscillation frequency. (**F**) Cross-correlation between $V_{\text {UCM}}(t)$ and $V_{\text {ORT}}(t)$. Negative values near zero (isochronous: *-0.02*, white: *-0.05*, pink: *-0.19*) indicate that task-relevant and task-irrelevant variance fluctuate independently, as expected from orthogonal geometric decomposition. The ratio operation obscures temporal structure: even when $V_{\text {UCM}}(t)$ and $V_{\text {ORT}}(t)$ individually exhibit distinct temporal patterns, their pointwise ratio carries the temporal signature of neither, so the time-varying ratio compounds the temporal collapse

To examine whether temporal structure manifests in the evolution of UCM variance ratios over time, we computed the instantaneous ratio $r_{\text {UCM}}(t)$ at each time point (Fig. 6A–C). Isochronous variability produced oscillatory fluctuations in $r_{\text {UCM}}(t)$ with period matching the imposed *0.1* Hz frequency ($mean\!=\!2.86$), reflecting phase-dependent coupling between task-space and UCM-space oscillations (Fig. 6A). White noise generated stochastic fluctuations around ($mean\!=\!8.58$ with no systematic temporal pattern (Fig. 6B). Pink noise exhibited similar stochastic fluctuations around $mean\!=\!8.83$ (Fig. 6C). Autocorrelation analysis of the ratio time series revealed no distinguishing features: white noise and pink noise showed similar autocorrelation structure despite individual variance components exhibiting clearly distinct temporal dependencies (Fig. 6D).

Power spectral density of $r_{\text {UCM}}(t)$ confirmed this similarity: white noise and pink noise ratio spectra were nearly indistinguishable, while the isochronous condition showed the expected spectral peak at *0.1* Hz (Fig. 6E). Cross-correlation between $V_{\text {UCM}}(t)$ and $V_{\text {ORT}}(t)$ was near zero for all conditions (isochronous: *-0.02*, white: *-0.05*, pink: *-0.19*), indicating largely independent fluctuations in task-relevant and task-irrelevant variance components, as expected from orthogonal geometric decomposition (Fig. 6F). The ratio operation itself obscures temporal structure: dividing two weakly correlated, independently fluctuating series discards the scaling, autocorrelation, and spectral properties of both. The time-varying ratio $r_{\text {UCM}}(t)$ therefore compounds the limitation of the time-averaged ratio, collapsing the two components into a single series that carries the temporal signature of neither.

### Per-trial indices: Geometric contrasts are negligible, temporal contrasts are large

Per-trial computation of all dependent variables ($N_{\text {trials}}\!=\!200$ per condition) enabled formal inferential testing across conditions (Table 1). Kruskal–Wallis tests indicated significant omnibus effects of condition for every variable examined (all $p\!<\!10^{-86}$; $\eta ^2_H$ ranging from *0.67* to *0.88*). The structurally informative result, however, lies in the pattern of pairwise effect sizes for the White vs. Pink contrast—the comparison most directly testing UCM sensitivity to temporal correlation, since these two conditions differ only in the temporal correlation structure of an otherwise matched stochastic process.

*Geometric indices yielded negligible effect sizes for the White vs. Pink contrast and did not survive Bonferroni correction.* The variance ratio $r_{\text {UCM}}$ (White: $8.69\!\pm \!1.66$; Pink: $8.67\!\pm \!0.83$; mean±SD across trials) gave Cliff’s $\delta \!=\!-0.04$ ($p\!=\!0.48$, Mann–Whitney $U\!=\!19{,}177$). The normalized variance difference $\Delta V_z$, the index most widely used in the synergy literature (Latash 2010 )(White: $1.23\!\pm \!0.09$; Pink: $1.24\!\pm \!0.05$), yielded the same statistical outcome ($\delta \!=\!-0.04$, $p\!=\!0.48$), since $\Delta V_z$ is a monotonic transformation of $r_{\text {UCM}}$ and the rank-based test is invariant under monotonic transformation. The component variances were similarly indistinguishable ($V_{\text {UCM}}$: $\delta \!=\!-0.07$, $p\!=\!0.23$; $V_{\text {ORT}}$: $\delta \!=\!+0.02$, $p\!=\!0.76$). By any standard interpretation, the White and Pink noise conditions are statistically indistinguishable on geometric indices.

*Temporal indices computed on the same trials yielded large pairwise effect sizes that easily survived Bonferroni correction, with autocorrelation and DFA carrying the strongest separation.* Lag-1 autocorrelation of the UCM-projected series produced essentially complete distributional separation (White: $0.003\!\pm \!0.088$; Pink: $0.451\!\pm \!0.112$; $\delta \!=\!-1.00$, $p\!=\!1.0\!\times \!10^{-66}$)—only one White trial out of $40{,}000$ White-Pink pairwise comparisons exceeded its Pink counterpart. DFA scaling of the UCM projection produced $\delta \!=\!-0.92$ (White: $\alpha _{\text {UCM}}\!=\!0.60\!\pm \!0.09$; Pink: $\alpha _{\text {UCM}}\!=\!0.88\!\pm \!0.12$; $p\!=\!7.0\!\times \!10^{-57}$). Per-trial spectral slope—which suffered from cross-trial aggregation—recovered a large but smaller separation when computed on single-trial projections ($\delta \!=\!+0.81$; $p\!=\!6.8\!\times \!10^{-45}$), corroborating but not driving the inferential conclusion. Equivalent patterns held for the ORT projection ($\rho _{\text {ORT}}(1)$: $\delta \!=\!-0.85$; $\alpha _{\text {ORT}}$: $\delta \!=\!-0.68$), with $\beta _{\text {ORT}}$ the lone exception at small magnitude ($\delta \!=\!-0.33$, $p\!=\!1.3\!\times \!10^{-8}$)—consistent with the broader pattern that spectral slope is the least robust of the three temporal indices in this regime.

This pattern—negligible effects on the per-trial geometric indices, large effects on the temporal indices—is a double dissociation in effect-size space: two processes indistinguishable on $r_{\text {UCM}}$, *Δ V*, and $\Delta V_z$ are perfectly separable on temporal grounds. That the three geometric indices yield identical outcomes follows from their being monotone transforms of one another, each collapsing the partition’s temporal evolution into a single number per trial. The ratio nonetheless remains useful: it sharply separates deterministic from stochastic regimes (isochronous vs. both stochastic conditions, all $|\delta |\!\ge \!0.93$), even though it cannot separate stochastic regimes differing only in temporal correlation.

The Isochronous vs. White and Isochronous vs. Pink contrasts produced large effects on every dependent variable (all $|\delta |\!\ge \!0.71$); these reflect the deterministic spatial structure imposed in the isochronous condition, a purely geometric effect. The critical inferential test of UCM’s insensitivity to temporal structure is therefore the White vs. Pink contrast, which the per-trial analyses resolve unambiguously.

**Table 1:** Per-trial statistical comparisons across conditions ($N_{\text {trials}}\!=\!200$ per condition). Values are mean ± SD. Kruskal–Wallis omnibus test (df *=\!2*). Pairwise Mann–Whitney *U* with Bonferroni correction ($\alpha _{\text {adj}}\!=\!0.0167$); Cliff’s *δ * is the effect size. The critical White-vs-Pink contrast (rightmost column) reveals negligible effects on geometric indices (top block) but large effects on temporal indices (bottom block)

Variable	Iso	White	Pink	KW *H* (*p*)	$\eta ^2_H$	W vs. P *δ * (*p*)
*Geometric indices*						
$V_{\text {UCM}}$ ($\times 10^{-3}$)	$1.7\!\pm \!0.3$	$10.0\!\pm \!1.3$	$10.0\!\pm \!0.2$	*400.0* ($<\!10^{-86}$)	*0.67*	*-0.07* (*0.23*)^ns^
$V_{\text {ORT}}$ ($\times 10^{-4}$)	$6.0\!\pm \!1.0$	$12.0\!\pm \!2.0$	$12.0\!\pm \!1.0$	*399.4* ($<\!10^{-86}$)	*0.67*	*+0.02* (*0.76*)^ns^
$r_{\text {UCM}}$	$2.92\!\pm \!0.69$	$8.69\!\pm \!1.66$	$8.67\!\pm \!0.83$	*399.6* ($<\!10^{-86}$)	*0.67*	*-0.04* (*0.48*)^ns^
*Δ V*	$1.82\!\pm \!0.21$	$2.52\!\pm \!0.08$	$2.53\!\pm \!0.04$	*399.6* ($<\!10^{-86}$)	*0.67*	*-0.04* (*0.48*)^ns^
$\Delta V_z$	$0.71\!\pm \!0.11$	$1.23\!\pm \!0.09$	$1.24\!\pm \!0.05$	*399.6* ($<\!10^{-86}$)	*0.67*	*-0.04* (*0.48*)^ns^
*Temporal indices*						
$\rho _{\text {UCM}}(1)$	$0.73\!\pm \!0.06$	$0.00\!\pm \!0.09$	$0.45\!\pm \!0.11$	*524.6* ($<\!10^{-113}$)	*0.88*	$\mathbf {-1.00}$ ($<\!10^{-65}$)^*^
$\rho _{\text {ORT}}(1)$	$0.98\!\pm \!0.01$	$0.00\!\pm \!0.10$	$0.24\!\pm \!0.14$	*494.6* ($<\!10^{-107}$)	*0.83*	$\mathbf {-0.85}$ ($<\!10^{-47}$)^*^
$\alpha _{\text {UCM}}$	$1.03\!\pm \!0.09$	$0.60\!\pm \!0.09$	$0.88\!\pm \!0.12$	*441.4* ($<\!10^{-95}$)	*0.74*	$\mathbf {-0.92}$ ($<\!10^{-56}$)^*^
$\alpha _{\text {ORT}}$	$1.98\!\pm \!0.11$	$0.61\!\pm \!0.09$	$0.76\!\pm \!0.12$	*461.6* ($<\!10^{-100}$)	*0.77*	$\mathbf {-0.68}$ ($<\!10^{-31}$)^*^
$\beta _{\text {UCM}}$	$-1.85\!\pm \!0.27$	$0.04\!\pm \!0.32$	$-0.63\!\pm \!0.40$	*486.2* ($<\!10^{-105}$)	*0.81*	$\mathbf {+0.81}$ ($<\!10^{-44}$)^*^
β_ORT_	−3.41±0.18	−1.72±0.20	−1.60±0.19	413.7(<10^−89^)	0.69	−0.33(<10^−7^)^*,†^

^ns^ not significant after Bonferroni correction. ^*^ significant at $\alpha _{\text {adj}}\!=\!0.0167$. ^†^ significant but small effect; the only temporal index where the White-vs-Pink magnitude is below the threshold for ‘large.” Cliff’s *δ *: $|\delta |\!<\!0.147$ negligible, $0.147\!-\!0.330$ small, $0.330\!-\!0.474$ medium, $\ge \!0.474$ large. Bold values indicate the large effects in the temporal block, in contrast to the negligible effects in the geometric block

## Discussion

The standard UCM summary cannot tell apart movements that differ only in their timing. Concretely, the time-averaged summaries by which UCM is conventionally reported—the ratio $\bar{r}_{\text {UCM}}$ and the *Z*-transformed synergy index $\overline{\Delta V_z}$—take equivalent values across motor variability with fundamentally distinct temporal structures. Per-trial testing gave a double dissociation for the critical white-versus-pink noise contrast: the geometric indices $V_{\text {UCM}}/V_{\text {ORT}}$, *Δ V*, and $\Delta V_z$ produced negligible effects (Cliff’s $\delta \!=\!-0.04$, $p\!=\!0.48$), while autocorrelation and DFA on the same trials produced large ones ($\rho _{\text {UCM}}(1)$: $\delta \!=\!-1.00$; $\alpha _{\text {UCM}}$: $\delta \!=\!-0.92$); per-trial spectral slope corroborated with a smaller effect ($\delta \!=\!+0.81$). That $\Delta V_z$—the index most commonly used to quantify task stabilization—is a monotonic transform of the ratio and yields identical outcomes, showing the limitation extends to every scalar formed by averaging the partition over time. The invariance is mathematical: the time average collapses the partition to a single number, thereby removing the temporal information. Two systems with identical time-averaged ratios may reflect completely different processes—active feedback at specific time constants, or passive accumulation of correlated noise—and the time-averaged indices assign them the same value.

The limitation of the time-averaged UCM summary has long been recognized. The decades-old effort toward “within-trial UCM” (Scholz et al. 2003) shows early developers knew that standard time-averaged reporting sets aside within-trial temporal information, and recent work has begun coupling geometric and temporal analyses (De et al. 2025; Grover et al. 2022). John et al. (2016) argued that redundant-task variability requires analyzing temporal structure alongside geometry; Martin et al. (2019) gave a process account of the causal contributors to UCM patterns; and Tommasino et al. (2021) noted that manifold methods miss how temporal fluctuations affect performance. None, however, quantified how invariant the time-averaged ratios are to different temporal structures. That quantification is our contribution: white, pink, and periodic dynamics yield ratios differing by only *2.6%* despite $\sim \!50\%$ differences in scaling exponents, so processes no control theory would equate are indistinguishable from time-averaged ratios yet separate cleanly once the projected components are analyzed in time. This limitation is orthogonal to Campolo et al.’s 2013 Riemannian solution to coordinate sensitivity because the two concern different sources of ambiguity: their method removes artifacts arising from the geometry of the chosen coordinates, whereas ours shows that even geometrically correct projections discard information about temporal organization. Both, therefore, limit mechanistic inference, but each requires a different remedy.

The inferential evidence presented here sharpens the limitation. Earlier work observed that temporal information is absent from the standard time-averaged summary, or called for process-level accounts of UCM structure (John et al. 2016; Martin et al. 2019; Tommasino et al. 2021). Our per-trial analysis quantifies the gap: two stochastic processes with statistically indistinguishable time-averaged partitions—across $V_{\text {UCM}}$, $V_{\text {ORT}}$, $V_{\text {UCM}}/V_{\text {ORT}}$, and $\Delta V_z$—separate completely on temporal grounds ($\rho _1$ reaches the maximum possible Cliff’s $\delta \!=\!-1.00$), even at $N\!=\!200$ trials/condition. The choice of summary thus governs what the analysis can see: a time-averaged synergy index assigns one value to processes that differ in mechanism, and *200* trials of statistical power leave that value unchanged. Mechanistic claims premised on $\Delta V_z$ alone—about active feedback regulation, redundancy exploitation, or anticipatory synergy adjustment—therefore rest on less than they appear to. Reporting temporal indices of the projected series alongside $\Delta V_z$ would expose such differences, yet it inherits an interpretive problem: the temporal behavior of a projected series reflects the geometric operation that produced it, and its meaning for control awaits independent validation. The synergy index and its temporal companions describe the data; establishing what either reveals about neural process remains a separate task.

Our simulations prescribe temporal structure directly, thereby isolating the aggregation step: holding spatial variance constant while varying only temporal structure shows that the time-averaged indices discard temporal dynamics, regardless of how they arise. This design choice is deliberate. Simulating specific controllers would entangle temporal and spatial properties—an optimal feedback controller lowers $V_{\text {ORT}}$ by its objective, thereby confounding the two—so prescribing structure establishes a necessary condition: time-averaged indices that stay invariant across temporal structures here will stay invariant when controllers and biomechanics interact in biological systems. That control architecture shapes temporal structure is well established: humans immediately correct goal-relevant deviations while letting goal-irrelevant ones persist (Dingwell et al. 2010), and long-range autocorrelations emerge from flexible control and shift with task demands (Vandamme et al. 2025). These signatures convey mechanistic information that the time-averaged ratio omits, and interpreting their presence in the projected components remains challenging.

Temporal analysis meets a limit of its own on the mechanics-versus-control problem, because passive biomechanics—frequency-dependent impedance, nonlinear damping, muscle-tendon dynamics—can produce rich temporal structure through purely passive means. Temporal and geometric methods, therefore, supply complementary constraints, each partial on its own. Passive systems yield fixed signatures set by physical parameters (exponential decay with $\tau \!=\!B/K$; resonances at $\omega _n\!=\!\sqrt{K/M}$) that hold constant across task contexts (Hogan 1984; Hunter and Kearney 1982; Kearney and Hunter 1982, 1990; Weiss et al. 1986), while active control adapts to task demands at $100\text {--}200$ ms timescales (Scott 2004; Shadmehr et al. 2010; Todorov and Jordan 2002), or shows multifractal regulation across scales (Eddy and Kelty-Stephen 2015; Fragaszy et al. 2024; Ihlen and Vereijken 2010; Kelty-Stephen et al. 2021; Kelty-Stephen and Mangalam 2022b). Task-dependent shifts in temporal structure implicate active control, so pairing the two analyses narrows the space of plausible mechanisms more than either does alone—with the identification of any given temporal signature still resting on the interpretive question set out above.

The same point applies, more briefly, to applications of UCM that test predictions of the Equilibrium Point Hypothesis (EPH). EPH holds that movements arise from shifts in centrally specified equilibrium positions with trajectory dynamics governed by spring-like neuromuscular properties, and it predicts specific temporal signatures—notably exponential convergence with time constants $\tau \!=\!B/K$ set by damping and stiffness (Feldman 2015; Latash 2019). Several studies have read high $V_{\text {UCM}}/V_{\text {ORT}}$ ratios during pointing and reaching as supporting EPH’s account of task-space stabilization with joint-space flexibility (Domkin et al. 2005; Scholz et al. 2000; Tseng et al. 2002). Our demonstration bears on these inferences as before: a high time-averaged ratio is consistent with EPH’s spring-like relaxation and equally with optimal feedback control, minimum-intervention strategies, or passive constraints aligned with task geometry, so the time-averaged index leaves the choice among them open. The temporal predictions of EPH (exponential decay at appropriate time constants, stiffness-dependent spectra) could in principle be examined on the projected components, and evidence that equifinality—a core EPH prediction—is violated in the redundant regime where UCM is applied (Zhou et al. 2014) makes such scrutiny pertinent. We, however, regard this line of validation as insufficient for reasons that extend beyond UCM. Threshold control specifies where equilibria lie and leaves unspecified how the system generates the temporally distinct coordination that real actions demand—the phase structure of reach, grasp, and lift, and the nonlinear adaptation of movement timing to distance, accuracy, and load (Mangalam and Stergiou 2026). Supplying that temporal organization requires machinery that EPH does not contain, so that recovering it presupposes a controller the hypothesis was meant to dispense with. A high UCM ratio interpreted through EPH therefore inherits a profound limitation: geometric evidence certifies spatial stabilization, while the temporal generation of movement—the property that discriminates among theories of control—lies outside both the time-averaged index and the equilibrium-point account it is taken to support.

This limitation is orthogonal to existing critiques of UCM. Those concern identifiability—partitioning outcomes depend on the choice of coordinates or reference frame (Sternad et al. 2010), on the Jacobian-estimation method (de Freitas and Scholz 2010), and on performance-variable and task definitions (Bennett et al. 2024). Ours holds even with all of these fixed and unambiguous: the time-averaged indices discard temporal structure, so identical time-averaged partitions arise from white, pink, or deterministic dynamics. Refinements of the geometric analysis leave this limitation in place, because it originates in the temporal collapse the summary performs, and it defines the boundary of what a geometric, time-averaged account can explain.

Motor variability also exhibits multifractal scaling—distinct exponents across scales, beyond monofractal *1/f* structure—documented in postural sway (Eddy and Kelty-Stephen 2015; Fragaszy et al. 2024; Ihlen and Vereijken 2010; Kelty-Stephen et al. 2021; Kelty-Stephen and Mangalam 2022b), gait (Mangalam et al. 2023; Wilson et al. 2023), effortful perception (Mangalam et al. 2020c, a, b; Mangalam and Kelty-Stephen 2020), and even reaching (Bell et al. 2019). The time-averaged indices are blind to these signatures (Kelty-Stephen and Mangalam 2022a, 2023; Mangalam and Kelty-Stephen 2022). Multifractal analysis, being a time-series method, applies directly to the projected trajectories, and recent work has begun coupling manifold partitioning with such dynamical analysis (De et al. 2025; Grover et al. 2022); the resulting exponents describe the projected series, and their mapping to a control process carries an enormous interpretive burden. The reporting practice thus preserves the same ambiguity—a multifractal system and a white-noise system can share identical time-averaged partitions while reflecting very different processes (Kelty-Stephen and Mangalam 2022a, 2023; Mangalam and Kelty-Stephen 2022). The clinical stakes follow: two patients with equal $V_{\text {UCM}}/V_{\text {ORT}}$ may differ in whether their variability reflects active regulation or passive filtering, a difference the time-averaged partition conceals, and one that temporal analysis can raise, but only a validated interpretation can settle.

Our simulation uses a simplified planar arm with prescribed temporal patterns. Biological motor control involves nonlinear, time-varying dynamics and coupled feedback, and biomechanical filtering may blur temporal signatures that are clean in simulation; whether autocorrelation, spectral, and scaling analyses retain sufficient sensitivity in empirical data is an open question. The structural property we rely on holds regardless of complexity: time-averaging collapses the partition and discards temporal ordering, while the projection transmits it. Prescribing structure directly is necessary here precisely because temporal and geometric properties are inextricably coupled in biological systems, and only simulation can isolate the aggregation step. The same aggregation logic applies to related decomposition methods. Principal component analysis (Jolliffe 2011), used in motor control to extract muscle synergies from pooled activity (d’Avella et al. 2003; Ting and Macpherson 2005; Tresch and Jarc 2009), and manifold learning (Lee and Verleysen 2007; Tenenbaum et al. 2000), used to identify low-dimensional neural manifolds for movement (Gallego et al. 2017), both characterize spatial structure and set temporal ordering aside unless applied to time-lagged coordinates (Broomhead and King 1986) or embedded in dynamical models (Brunton et al. 2016). The neural-manifold literature draws the parallel especially close, partitioning population activity into output-potent and output-null subspaces in direct analogy to the task-relevant and task-irrelevant partition of UCM. Finally, a note on the unit of analysis: the per-trial temporal indices (Table 1) are computed on single-trial projections, while the cross-trial series $V_{\text {UCM}}(t)$, $V_{\text {ORT}}(t)$ are variances across trials at each instant. Both reveal the dissociation, and empirical studies should prefer per-trial indices, which admit inferential statistics across trials and capture exactly what time-averaging discards.

The core issue is that a high ratio establishes *that* coordination is task-structured—a real finding, since $V_{\text {UCM}}\!>\!V_{\text {ORT}}$ varies with task demands and holds only for some systems—while the same value arises equally from active feedback, passive mechanics, anatomical constraints, or filtered noise. Testing $V_{\text {UCM}}\!>\!V_{\text {ORT}}$ is a legitimate operationalization of a synergy, and we accept that inferential logic; our narrower point is that the time-averaged index alone leaves undetermined *which* process produced the structure, which is often the substantive question. High $V_{\text {UCM}}/V_{\text {ORT}}$ in multi-finger (Latash et al. 2001; Scholz et al. 2003; Tillman and Ambike 2018) and postural (Reimann and Schöner 2017; Scholz and Schöner 1999) tasks have been interpreted as active force or center-of-mass stabilization without a temporal test, and the geometric result there carries a process-level claim that exceeds what the method can deliver. Temporal analysis of the projected components adds information, and that information demands its own interpretation before it can settle the question; the geometric method retains the limitation regardless.

The time-averaged indices have a well-defined scope. Within it, the ratio separates deterministic motion from stochastic motion (Fig. 3J). Two further analyses extend that reach: time-resolved analysis of $V_{\text {UCM}}(t)$ and $V_{\text {ORT}}(t)$ separates deterministic, white, and pink structure (Fig. 4), and phase-resolved UCM characterizes the feedforward, anticipatory component of control (Goodman and Latash 2006; Olafsdottir et al. 2005; Shim et al. 2005). One distinction falls outside this scope: distinguishing stochastic processes that share instantaneous covariance—active from passive, or one correlation structure from another. The time-averaged ratio assigns the same value to such processes, and pursuing the distinction through the projected components reopens the interpretive question of what their temporal properties signify.

A full account of control draws on both geometric and temporal analysis of the same data. The two share common ground: dynamical systems theory is itself geometric—flows, fixed points, and limit cycles are geometric objects, and fractal and multifractal analyses rest on Mandelbrot’s fractal geometry—so the operative contrast lies between a static time-averaged summary and a temporally resolved analysis of the same projected components. The partition shows the spatial distribution of variability, contingent on the investigator’s modeling choices (performance variables, coordinate frame, Jacobian estimation), and temporal analysis of the projected components proposes candidate generating processes whose identification depends on validating what the temporal properties mean. Progress requires studies that pair the two and confront that validation directly: testing whether feedback systems exhibit the predicted autocorrelation time constants, whether clinical populations differ in geometric or temporal structure, or both, and whether equilibrium-point and optimal-control accounts reproduce the observed spectral and long-range correlation properties (cf. Mangalam et al. 2023). The move from asking “Is variability organized?” to asking “What process organizes it?” sets the agenda; the present work shows that geometric partitioning alone leaves the second question open, and that answering it will demand its own theoretical foundation.

## Conclusion

Uncontrolled Manifold (UCM) analysis partitions motor variability into task-relevant and task-irrelevant components by projecting deviations in elemental variables onto Jacobian-defined subspaces. Our central result concerns the practice of reporting that partition through time-averaged summaries—the ratio $\bar{r}_{\text {UCM}}$ and the synergy index $\overline{\Delta V_z}$. These summaries remain insensitive to temporal structure: white and pink noise yield statistically indistinguishable values across orders of magnitude in autocorrelation and scaling. The insensitivity originates in the time-averaging step; the projection transmits temporal information to $V_{\text {UCM}}(t)$, $V_{\text {ORT}}(t)$, and the per-trial projections, where its meaning for control remains an open question. The time-averaged ratio separates deterministic from stochastic regimes while assigning matched values to stochastic processes that share instantaneous covariance. A high ratio thus establishes that variability is organized along task-relevant directions—a real and useful finding—and leaves the generating process—whether active control, passive mechanics, or biomechanical filtering—undetermined.

The temporal information that time-averaging discards survives in the single-trial and time-resolved projections, and this survival marks the frontier that the method leaves unresolved. Autocorrelation, detrended-fluctuation, spectral, and multifractal analyses apply to the projected components, yielding exponents and correlation structure; the meaning of those quantities for control is a separate matter, since the projected series inherit the geometry of the Jacobian and task variables, and reading control from their temporal behavior requires theoretical and empirical justification. UCM describes where variability lies relative to task demands. The processes generating that variability over time lie outside its reach, and locating them calls for methods whose interpretation has been established independently. This is the standing limitation of a geometric, time-averaged account of motor control: it maps the spatial organization of variability with precision while leaving the dynamical origin of that organization to a theory yet to be built.

## Data Availability

No datasets were generated or analysed during the current study.
